# Proliferation and Invasion of Melanoma Are Suppressed by a Plant Protease Inhibitor, Leading to Downregulation of Survival/Death-Related Proteins

**DOI:** 10.3390/molecules27092956

**Published:** 2022-05-05

**Authors:** Camila Ramalho Bonturi, Bruno Ramos Salu, Camila Nimri Bonazza, Rita de Cassia Sinigaglia, Tiago Rodrigues, Miryam Paola Alvarez-Flores, Ana Marisa Chudzinski-Tavassi, Maria Luiza Vilela Oliva

**Affiliations:** 1 Departamento de Bioquímica, Universidade Federal de São Paulo (UNIFESP), São Paulo 04044-020, Brazil; camilabntr@gmail.com (C.R.B.); bruno_salu@hotmail.com (B.R.S.); c_bonazza@hotmail.com (C.N.B.); 2 Electron Microscopy Center, Universidade Federal de São Paulo (UNIFESP), São Paulo 04044-020, Brazil; rita.sinigaglia@unifesp.br; 3 Centre for Natural and Human Sciences, Universidade Federal do ABC (UFABC), Santo André 09210-580, Brazil; tiago.rodrigues@ufabc.edu.br; 4 Centre of Excellence in New Target Discovery (CENTD), Instituto Butantan, São Paulo 05503-900, Brazil; miryam.flores@butantan.gov.br (M.P.A.-F.); ana.chudzinski@butantan.gov.br (A.M.C.-T.)

**Keywords:** EcTI, melanoma, metastasis, protease inhibitor, skin cancer

## Abstract

Cell adhesion and migration are crucial for cancer progression and malignancy. Drugs available for the treatment of metastatic melanoma are expensive and unfit for certain patients. Therefore, there is still a need to identify new drugs that block tumor cell development. We investigated the effects of *Enterolobium contortisiliquum* trypsin inhibitor (EcTI), a protease inhibitor, on cell viability, cell migration, invasion, cell adhesion, and cell death (hallmarks of cancer) in vitro using human melanoma cells (SK-MEL-28 and CHL-1). Although EcTI did not affect non-tumor cells, it significantly inhibited the proliferation, migration, invasion, and adhesion of melanoma cells. Investigation of the underlying mechanisms revealed that EcTI triggered apoptosis and nuclear shrinkage, increased PI uptake, activated effector caspases-3/7, and produced reactive oxygen species (ROS). Furthermore, EcTI disrupted the mitochondrial membrane potential, altered calcium homeostasis, and modified proteins associated with survival and apoptosis/autophagy regulation. Acridine orange staining indicated acidic vesicular organelle formation upon EcTI treatment, demonstrating a cell death display. Electronic microscopy corroborated the apoptotic pattern by allowing the visualization of apoptotic bodies, mitochondrial cristae disorganization, and autophagic vesicles. Taken together, these results provide new insights into the anti-cancer properties of the natural EcTI protein, establishing it as a promising new therapeutic drug for use in melanoma treatment.

## 1. Introduction

Malignant melanoma is a highly aggressive tumor that typically arises from melanocytic nevus or the transformation of melanocytes. Patients with an early diagnosis are more likely to undergo cure-rendering treatment; however, the cancerous cells have the potential to invade adjacent organs within a short period [1]. Several drugs, such as target treatments, immunotherapy, or a combination of both, have been approved by the Food and Drug Administration over the past few years. Currently approved therapies include multiple strategies that depend on the melanoma stage, its location in the body, and the patient’s health. Nonetheless, these treatments face numerous challenges, including undesirable adverse effects that may lead to a severe immune response or the death of healthy cells. Tumor cell resistance is also common, increasing the difficulty of discovering new therapies [2]. Natural antitumor products are largely applied in preclinical studies and could be used as anti-melanoma agents if they exhibit low toxicity in normal cells, such as diterpene compounds isolated from *S. miltiorrhiza* roots and the polyphenol of *Olea europaea* leaves [3,4]. Moreover, many Kunitz-type inhibitors on different classes of proteolytic enzymes have been described as having profound effects on hemostasis and tumors, suggesting possible anticoagulant, anti-inflammatory, and antimetastatic potential [5,6,7,8]. This study was developed to provide new insights into melanoma treatment since plant proteins are showing success in several recent approaches [1,2,3,4,5,6,7,8]. We focused on a plant-derived protein with anti-cancer properties, the *Enterolobium contortisiliquum* trypsin inhibitor (EcTI), a 20 kDa inhibitor, isolated from *Enterolobium contortisiliquum* seeds. This protein exhibits inhibitory activity against a variety of enzymes [9,10]. EcTI has shown promising effects against a variety of cancer cells, including gastric cancer cell lines (Hs746T and MKN28), without any sensitive effect on fibroblasts isolated from amniotic fluid [9]. EcTI also interferes with colorectal cells (HCT116 and HT29), breast cancer cells (SkBr-3 and MCF-7), and leukemia cells (K562 and THP-1), with no affected target in human mesenchymal stem cells [11]. EcTI also affected glioblastoma cell lines (U87) and potentiated the anti-cancer effect of a stem cell in a deadly brain tumor [12]. It was recently reported that EcTI, when bound to collagen I, triggers several pathways that decrease cell viability and the invasion of triple-negative breast cancer cells [13]. The aim of this study was to better understand the mechanisms by which EcTI exerts its anti-cancer properties, analyzing apoptosis, autophagy, and other characteristics of tumor cells, including migration, adhesion, and invasion.

## 2. Results

### 2.1. EcTI Affected the Viability and Proliferation of Melanoma Cells

**Figure 1:**
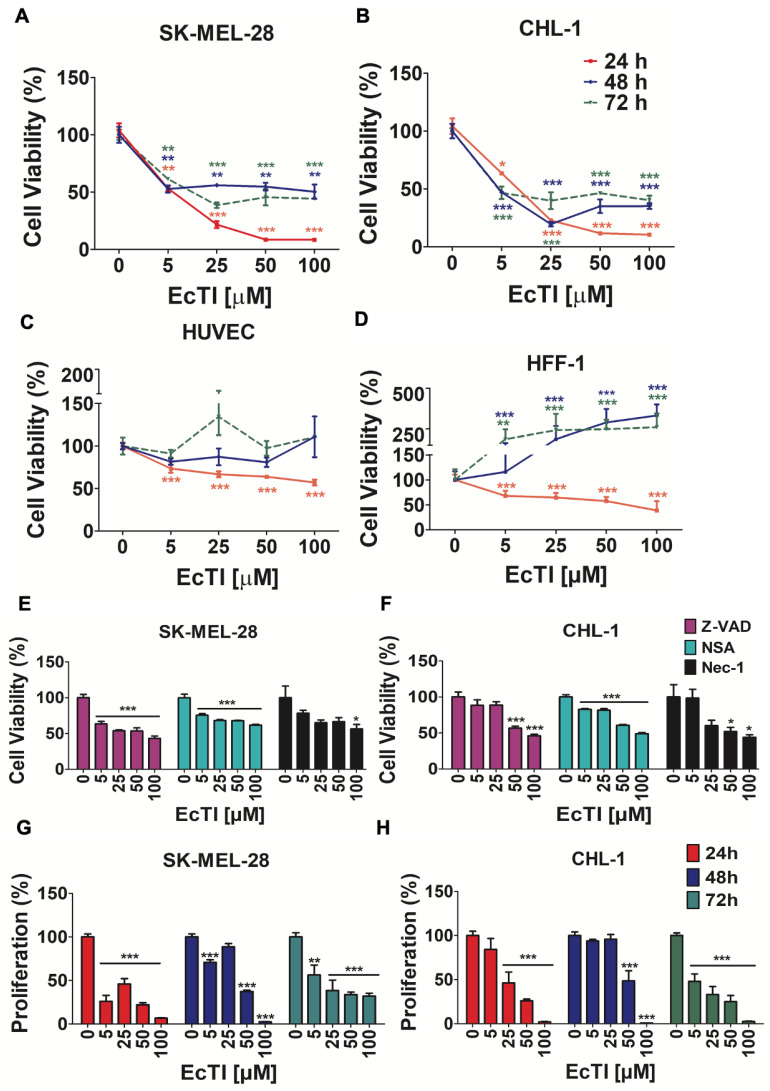
Cell viability and proliferation. Cell viability of SK-MEL-28 (**A**), CHL-1 (**B**), and non-tumorigenic HUVEC (**C**) and HFF-1 (**D**) was measured with increased concentrations of EcTI (5, 25, 50, and 100 µM) after 24, 48, and 72 h of treatment. Co-treatment with apoptotic and necrotic inhibitor was evaluated after 24 h of incubation with Z-VAD (20 µM), NSA (0.2 µM), and Nec-1 (5 µM) in SK-MEL-28 (**E**) and CHL-1 (**F**) cells and demonstrated the alteration of Z-VAD and Nec-1, especially. Proliferation assays were estimated by BrDU uptake in SK-MEL-28 (**G**) and CHL-1 (**H**) cells after 24–72 h of EcTI treatment. Percentage values were normalized to control cells (0). The bars represent means and SDs. Significance reflected: * *p* < 0.05, ** *p* < 0.005, and *** *p* < 0.0005.

Evaluation of cell viability is essential for screening the cytotoxicity of compounds in tumor cells [14]. EcTI (100 µM, 24 h) (Figure 1) remarkably decreased the viability of both melanoma cells to a similar extent as the positive control, Triton X-100 (0.01%) (Figure S7, in the Supplementary Materials). These effects were preserved even after 72 h, indicating the resistance of EcTI after long exposure to cellular proteases (Figure 1A,B). Although, after 24 h, EcTI reduced the viability of HFF-1 (IC_50_: 70 µM) and HUVEC cells (IC_50_: 108.8 µM; Figure 1C,D), after 48 h, the effects were no longer observed. On the other hand, the efficacy of inhibiting the viability of the melanoma cells, SK-MEL-28 (IC_50_: 32.08 µM) and CHL-1 (IC_50_: 34.64 µM), was higher than that presented in the non-tumorigenic cells (keratinocytes, HACAT (IC_50_: 131.81 µM) and melanocytes, melan-a (IC_50_: 69.73 µM), Figure S8), and the effects were maintained even after 48 and 72 h. To confirm this, we evaluated the selectivity index, which measures the cytotoxicity and tumorigenic activity of a compound by the ratio of non-tumorigenic/tumorigenic cells. The selectivity index (SI) for SK-MEL-28 and CHL-1 compared to HUVEC was 3.39 and 3.14, in comparison to HFF-1 was 2.18 and 2.02, compared to HACAT was 4.1 and 3.8, and compared to melanocytes was 2.17 and 2.01, respectively (Figure S8). In both cases, SI > 2, which shows the high toxicity toward the melanoma cell lines, but low toxicity toward the normal cell lines, HUVEC and HFF-1, corroborating the cell death assay (flow cytometry), in which no alteration at 25 and 50 µM of EcTI was observed, with a slight increase in necrosis at 100 µM of EcTI (2.5% of necrosis in control to 6.6% of necrosis after EcTI treatment, Figure S9).

Co-treatment with apoptosis/necroptosis inhibitors helped to clarify the EcTI action. Z-VAD irreversibly binds to caspases, protecting cells from apoptosis, while Nec1 and NSA prevent cells from necroptosis [15]. Thus, Z-VAD/EcTI co-treatment increases cell viability compared to EcTI treatment alone in both SK-MEL-28 and CHL-1 cells (Figure 1E,F, respectively), due to the inhibition of caspase activity. Although the main change in EcTI efficacy was in the presence of Nec-1, EcTI effects were slightly affected by NSA, indicating a contribution of necroptosis. Nec-1 blocks necrosis through ROS induction and caspase-8 activation, which may stimulate caspase-3/7, indicating the need for further investigation of this pathway. With respect to cell proliferation, EcTI significantly reduced this event, by approximately 50% or more at 50 and 100 µM, in all periods analyzed (24–72) (Figure 1G,H).

### 2.2. EcTI Impaired Adhesion, Migration, and Invasion of Melanoma Cells

**Figure 2:**
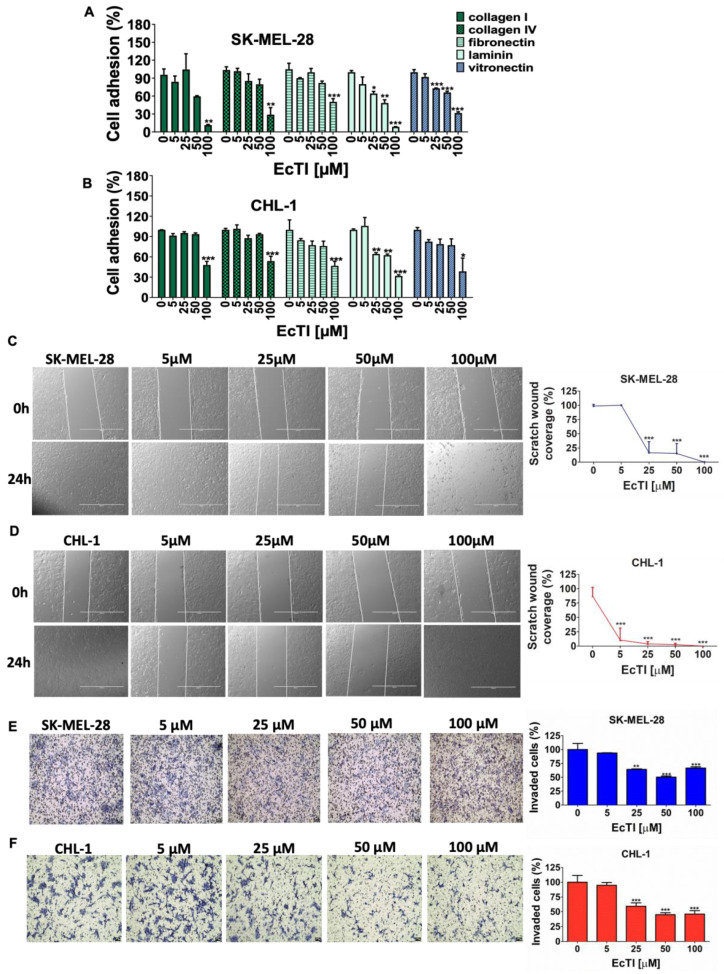
Cell adhesion, migration, and invasion. The effects of EcTI on cell adhesion of SK-MEL-28 (**A**) and CHL-1 (**B**) cells after pre-incubation of matrix molecules such as collagen I and IV, fibronectin, laminin, and vitronectin in the presence of increasing concentrations of EcTI (5, 25, 50, and 100 µM) show reduced interactions between EcTI and all matrix molecules, particularly laminin and vitronectin. Cell migration was assessed using the scratching assay after EcTI incubation on melanoma cells; a pronounced effect of above 70% was observed in SK-MEL-28 (**C**) and CHL-1 (**D**). The invasion assay was performed using the Boyden chamber technique with different concentrations of EcTI for 24 h; a superior reduction (25%) in the invasion capability of SK-MEL-28 (**E**) and CHL-1 (**F**) cells was observed (scale bar 20 µm). All percentage values were normalized to control cells (“0”). The bars represent means and SDs. Significance was considered as * *p* < 0.05, ** *p* < 0.005, and *** *p* < 0.0005.

Actin cytoskeleton interactions are essential for metastasis formation because tumor cells migrate, invade, and then spread to surrounding tissues. Thus, cell adhesion proteins are required to promote these processes, and inhibitors are used as potential anti-cancer tools [16,17,18]. To verify the effect of EcTI on cell adhesion, an important hallmark of cancer, EcTI (100 µM) interfered with adhesion mediated by collagen I, collagen IV, fibronectin, laminin, and vitronectin. Importantly, the reduction in SK-MEL-28 adhesion to laminin and vitronectin (Figure 2A) and CHL-1 to laminin (Figure 2B) was remarkable.

Since blocking cell adhesion prevents cell migration, the strong effect of EcTI on the blocking of cell migration was not a surprise. Here, we show that non-treated cells migrated faster to close the gap of a scratch in the cell monolayer melanoma treated cells: at doses of 25–100 µM for SK-MEL-28 and 5–100 µM for CHL-1, EcTI inhibited over 70% of migration (Figure 2C,D). At a dose of 100 µM, cells were detached (Figure S10), demonstrating EcTI as an inducer of apoptosis, as previously suggested by co-treatment data with death/necroptosis inhibitors.

Invasion is a pathological process that may contribute to the dissemination of malignant cells throughout different bodily tissues. EcTI impaired the invasive ability of melanoma cells by more than 30% (Figure 2E,F, respectively), demonstrating a positive outcome in preventing cancer progression.

### 2.3. EcTI Provoked Chromatin Condensation and PI Uptake

**Figure 3:**
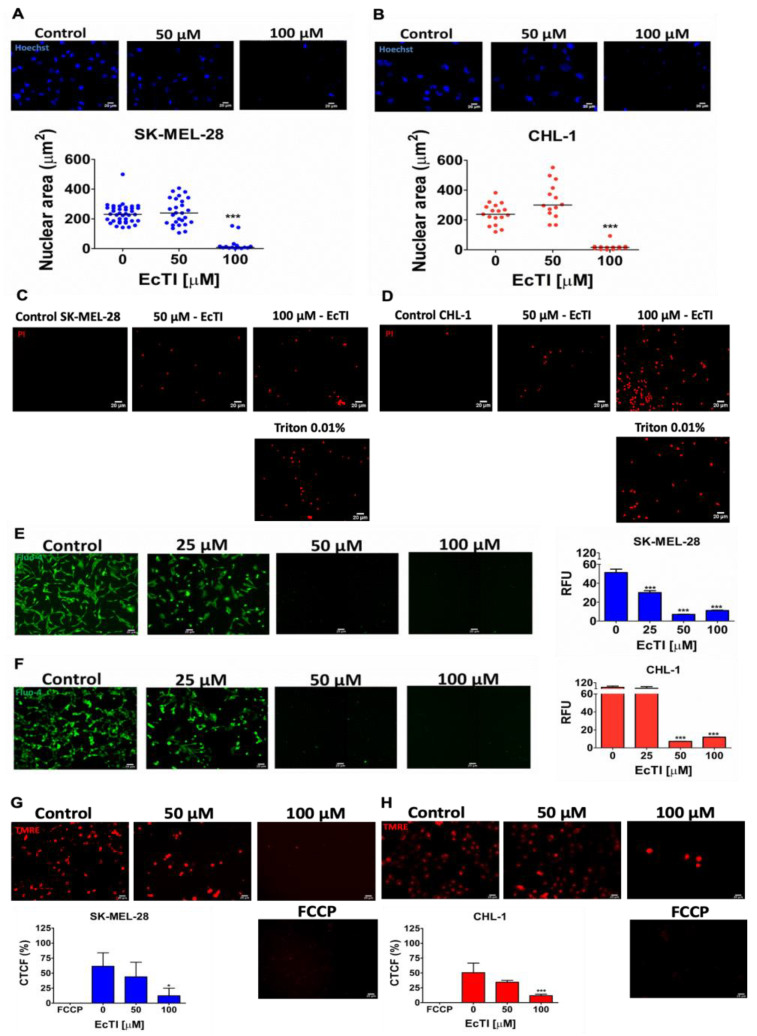
Nuclear morphology, PI uptake, calcium release, and mitochondrial membrane potential. Cell morphology to detect nuclear alteration in SK-MEL-28 cells (**A**) and CHL-1 (**B**) was determined using Hoechst staining after EcTI incubation for 24 h; pyknotic nuclei and nuclear shrinkage were observed after incubation with 100 µM of EcTI. Loss of membrane integrity was calculated using PI fluorophore after incubation with 50 and 100 µM of EcTI; extra fluorescence cells (particularly after incubation with 100 µM of EcTI) were seen in SK-MEL-28 (**C**) and CHL-1 (**D**) (scale bar 20 µm). Triton X-100 (0.01% *v*/*v*) was used as a positive control of cell death. The effect of EcTI on calcium release was observed through fluorescence microscopy and using a microplate reader in the FLUO-4AM direct assay. The compound provoked a decline in melanoma cell fluorescence, as seen via microscopy after incubation with 25, 50, and 100 µM of EcTI ((**E**) SK-MEL-28 and (**F**) CHL-1, scale bar 10 mm). The effect was confirmed using the microplate reader at 485 nm excitation/530 nm emission and expressed using a relative fluorescence unit (RFU). Mitochondrial membrane potential was also analyzed through fluorescence with TMRE staining; a disruption in mitochondria stimulated by pore opening in SK-MEL-28 (**G**) and CHL-1 (**H**) was observed (scale bar 20 µm). FCCP: 50 µM (carbonyl cyanide 4-((trifluoromethoxy) phenylhydrazone) and CTCF: integrated density— (area of selected cell × mean fluorescence of FCCP readings). The bars represent means and SDs. Significance was considered as * *p* < 0.05, ** *p* < 0.005, and *** *p* < 0.0005.

Due to necrotic or apoptotic events, the nuclei of cells exhibit morphological changes that can be useful indicators of cell death [19]. SK-MEL-28 (Figure 3A) and CHL-1 (Figure 3B) exhibited dramatic changes in their nuclear sizes, indicating nuclear condensation and pyknotic nuclei induced by EcTI treatment. The cell death process includes the loss of cell membrane integrity, which can be observed by PI incorporation. Therefore, we analyzed the difference in membrane integrity after EcTI treatment using the small nuclear cell-impermeant fluorophore PI. The loss of cell membrane integrity was observed, especially at 100 µM (Figure 3C,D), suggesting that EcTI affects cell death events.

### 2.4. EcTI Modified Cytosolic Ca^2+^ Levels, Mitochondrial Membrane Potential, and ROS Production

Apoptosis and necrosis are involved in the disruption of Ca^2+^ homeostasis, triggering signaling to a variety of mechanisms, such as autophagy, necrosis, and apoptosis [20,21]. EcTI impaired intracellular calcium levels in both cell lines (Figure 3E,F). Alterations in mitochondrial membrane permeability are associated with apoptotic or necrotic cell death due to BAX oligomerization (MOMP, mitochondrial outer membrane permeabilization) or mitochondrial permeability transition (MPT). The disruption of cellular Ca^2+^ homeostasis could trigger mitochondrial permeabilization, which is intrinsically related to the dissipation of mitochondrial membrane potential and cell death [21]. EcTI (100 µM) decreased the TMRE fluorescence of both human melanoma cells (Figure 3G,H), suggesting that the protease inhibitor EcTI induces mitochondrial permeabilization.

**Figure 4:**
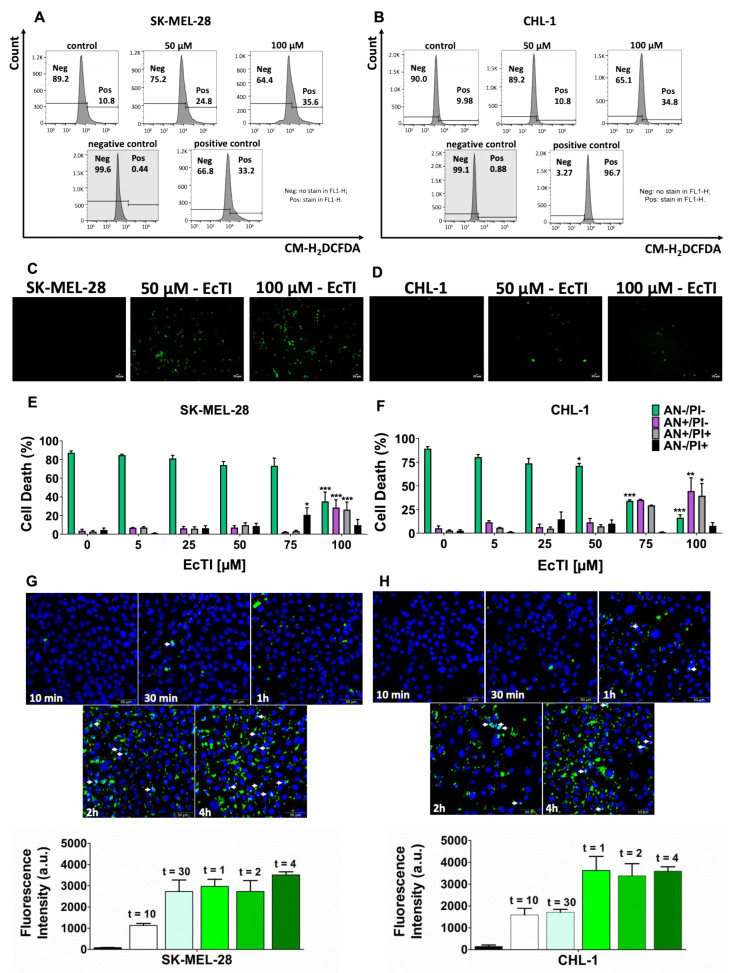
ROS production, caspase-3/7 activity, cell death, and EcTI internalization. Intracellular reactive oxygen species (ROS) were measured using 2,7-dichlorodihydrofluorescein diacetate (CM-H_2_DCFDA), and fluorescence was measured using flow cytometry with an FL1-H channel on a BD Accuri C6 flow cytometer (BD, Los Angeles, CA, USA) and Flow Jo 10 software. EcTI induced ROS production in both human melanoma cells lines, (**A**) SK-MEL-28 and (**B**) CHL-1, as seen in the histograms. The negative control was melanoma cells without CM-H_2_DCFDA staining, and the positive control was 500 µM H_2_O_2_. The *y*-axis represents cell counts, and the *x*-axis represents the intensity of green fluorescence (ROS). Neg: no stain in FL1-H; Pos: stain in FL1-H. The action of EcTI on caspase-3/7 activity was demonstrated using DEVD polypeptide substrate after EcTI incubation for 24 h; an increase in cell fluorescence after treatment with 100 µM of EcTI in both SK-MEL-28 (**C**) and CHL-1 (**D**) was observed. Melanoma cell death in SK-MEL-28 (**E**) and CHL-1 (**F**) was investigated through flow cytometry, using fluorophores annexin-FITC (AN) and propidium iodide (PI), after EcTI incubation (5, 25, 50, 75, and 100 µM) for 24 h. AN^−^/PI^−^ indicates viable cells; AN^+^/PI^−^ indicates early apoptotic cells; AN^+^/PI^+^ indicates early apoptotic cells; AN^−^/PI^+^ indicates necrotic cells. The control is represented by “0”. The effect of EcTI on cell internalization after 10 min, 30 min, 1 h, 2 h, and 4 h ((**G**) SK-MEL-28 and (**H**) CHL-1) was analyzed via EcTI conjugation with AlexaFluor488 and nuclear Hoechst staining, where compound internalization became gradually visible over time. The bars represent the mean and SD. Significance was considered as * *p* < 0.05, ** *p* < 0.005, and *** *p* < 0.0005.

The production of reactive oxygen species (ROS) depends on levels of Ca^2+^ and can regulate the opening of the mitochondrial permeability transition pore. The accumulation of ROS is linked to DNA damage, which plays an important role in cell dysfunction and increased disease states, such as apoptosis [22,23]. EcTI increased ROS production, especially at 100 µM, in both melanoma cell lines, as seen in the histogram plots (Figure 4A,B and Figure S11), corroborating nuclear size alteration, mitochondrial dysfunction, and cell death.

### 2.5. EcTI Promotes Caspase-Dependent Apoptosis in Melanoma Cells

Several morphological changes in cells are indicative of the triggering of cell death. The activity of caspases has been related to apoptosis, although investigators have observed an apoptosis-independent pathway [15]. Although caspase-3/7 activity seemed to be more expressive in the SK-MEL-28 cells than in the CHL-1 cells, EcTI increased the activity of caspase-3/7 in both human melanoma cell lines, demonstrating the essential role of the protease during apoptosis (Figure 4C,D and Figure S12).

Under pathological conditions, morphological alterations can occur, such as DNA degradation, nuclear condensation, activation of caspases, loss of plasma membrane asymmetry, membrane blebbing, and phospholipid phosphatidylserine exposure. Apoptosis was evaluated using annexin V-FITC (AN) and propidium iodide (PI) [24]. Double staining was used to verify different stages of cell death: at 100 µM, SK-MEL-28 showed a greater than 50% reduction in the number of viable cells, a 25% increase in early apoptosis, and an approximately 24% increase in late apoptosis, while CHL-1 displayed more than 70% inhibition of cell survival, increased by 31% during early apoptosis and 37% during late apoptosis. Thus, EcTI significantly decreased cell survival by promoting apoptosis in both SK-MEL-28 and CHL-1 cell lines (Figure 4E,F).

### 2.6. EcTI Is Internalized by Melanoma Cells

The cell membrane is a barrier that simultaneously protects the cell from pathogens and hinders the effectiveness of drugs; therefore, the cellular internalization of EcTI is relevant to exerting its anti-cancer effect. Uptake of EcTI-AlexaFluor488 at different periods showed a large increase in intracellular fluorescent intensity over time in both melanoma cell lines (Figure 4G,H).

### 2.7. The Activity of EcTI Is Dependent on Apoptotic/Autophagic-Related Proteins

**Figure 5:**
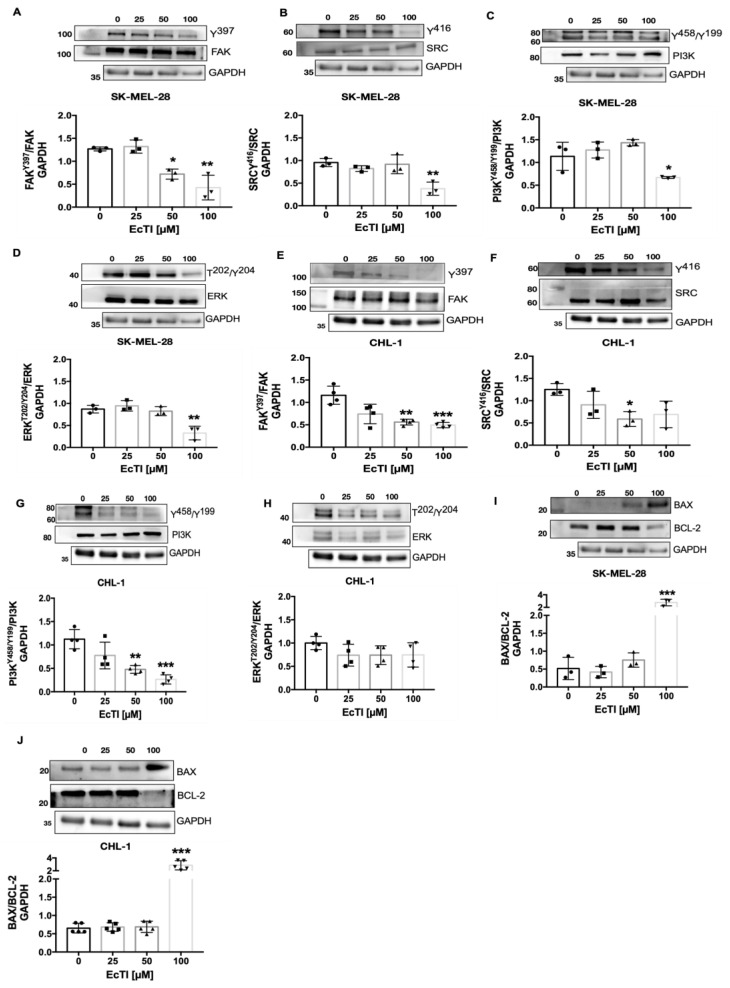
Survival and apoptotic-related proteins. The action of EcTI on different proteins related to cell survival and apoptosis in human melanoma cells is depicted. The proteins in panels (**A**–**H**) are associated with cell proliferation, migration, and invasion, while those in panels (**I**,**J**) are associated with cell death. EcTI interfered with FAK (**A**), SRC (**B**), PI3K (**C**), and ERK (**D**) in SK-MEL-28 cells and with FAK (**E**), SRC (**F**), and PI3K (**G**) in CHL-1 cells; however, ERK (**H**) was not affected in CHL-1 cells. Furthermore, EcTI affected the expression of the cell death protein BAX/BCL-2 in both SK-MEL-28 (**I**) and CHL-1 (**J**) cells. The graphs are provided in arbitrary units (a.u.), and all proteins except for the phospho-total ratio were normalized with the housekeeping protein, GAPDH. The control is represented by “0”. The bars represent means and SDs. Significance was considered as * *p* < 0.05, ** *p* < 0.005, and *** *p* < 0.0005.

FAK and SRC are kinases that promote cancer growth and progression, which play a role in activating downstream cellular signaling pathways required for the survival, proliferation, adhesion, and invasion of cells [16]. ERK and PI3K also participate in the regulation of tumor invasion and metastasis [17]. EcTI (100 µM) decreased FAK, SRC, and PI3K expression in SK-MEL-28 and CHL-1 (Figure 5A–H). ERK was also affected in SK-MEL-28 cells, while its expression was not affected in CHL-1 cells (Figure 5D,H), indicating a different pattern for EcTI treatment upon both lineages.

**Figure 6:**
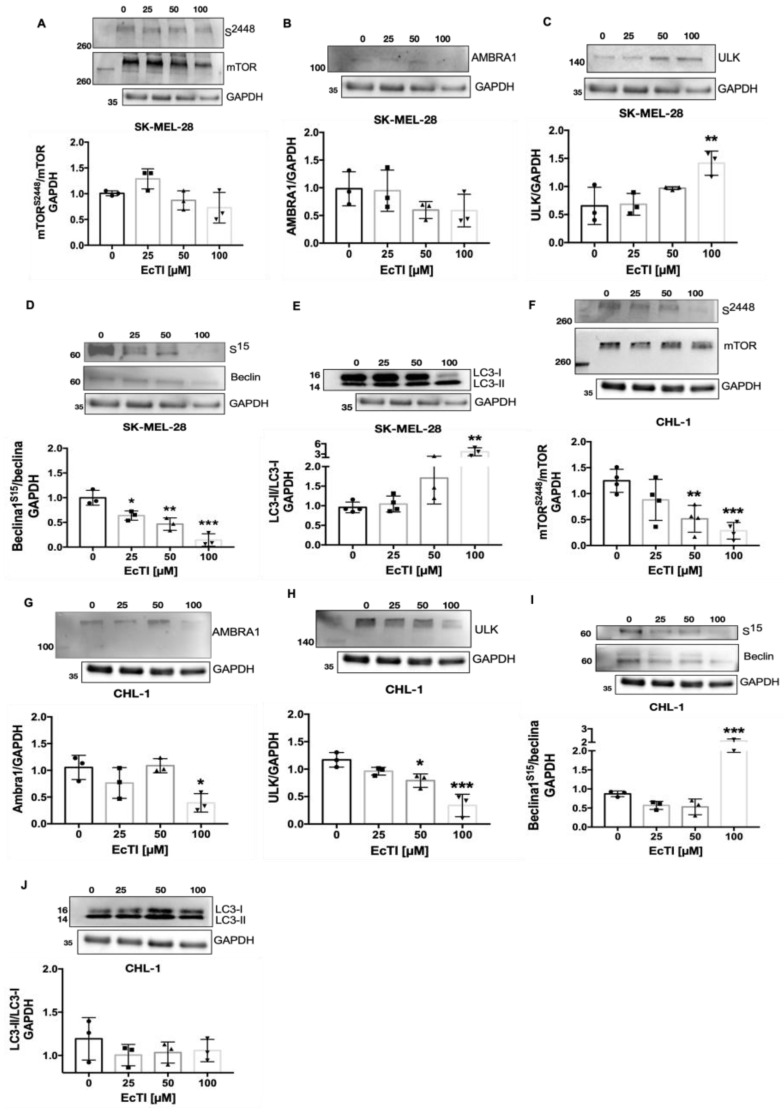
Autophagy-related proteins. The action of EcTI on different proteins related to the autophagic process is depicted in panels (**A**–**J**). EcTI affected autophagy protein levels. Although no alterations were observed in mTOR (**A**) and AMBRA (**B**), EcTI impaired ULK (**C**), BECLIN (**D**), LC3-II/I (**E**) in SK-MEL-28 cells. In CHL-1 cells, EcTI induced changes in mTOR (**F**), AMBRA (**G**), ULK (**H**), and BECLIN (**I**); however, no alteration was visible on LC3-II/I (**J**). The graphs are provided with arbitrary units (a.u.), and all proteins except for the phospho-total ratio were normalized with the housekeeping protein, GAPDH. The control is represented by “0”. The bars represent means and SDs. Significance was considered as * *p* < 0.05, ** *p* < 0.005, and *** *p* < 0.0005.

BAX and BCL-2 are associated with cell death and are responsible for controlling MOMP, caspase activation, and apoptosis [25]. The BAX/BCL-2 ratio significantly increased, suggesting a significant increase in the expression of the proapoptotic protein BAX and diminished expression of the anti-apoptotic protein BCL-2 (Figure 5I,J). Since cell death is characterized in several stages by distinct features such as changes in proteins linked to apoptosis and autophagy, we studied the impact of EcTI on autophagy-related proteins. The induction of autophagy can be triggered by intra- and extracellular stimuli, such as mitochondrial damage and oxidative stress, engaging proteins such as ULK1, AMBRA1, BECLIN, and LC3 in autophagosome formation [25,26]. In the SK-MEL-28 lineage under EcTI treatment (100 µM), no change in mTOR and AMBRA expression was observed, whereas ULK expression was remarkably increased. BECLIN expression declined, accompanied by an increase in the LC3-II/LC3-I ratio. In the CHL-1 lineage, mTOR and AMBRA were downregulated in a dose-dependent manner, similar to ULK. On the other hand, BECLIN was upregulated, with no effect observed in LC3 (Figure 6A–J).

### 2.8. EcTI Stimulated Acidic Vesicular Organelle Formation

**Figure 7:**
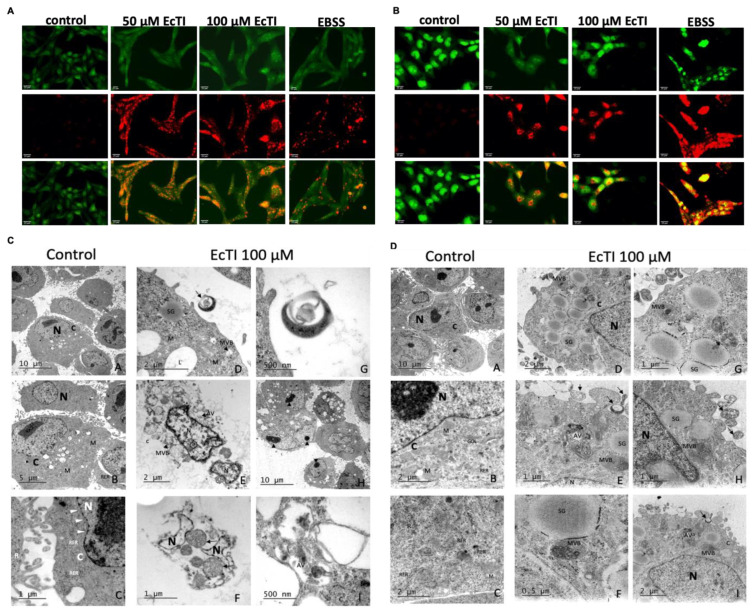
Acidic vesicular organelle formation and transmission electron microscopy. Cells were incubated with 50 and 100 µM EcTI for 24 h or EBSS medium for 3 h (positive control). After treatment, SK-MEL-28 (**A**) and CHL-1 (**B**) cells were stained with 0.5 μg/mL acridine orange (AO) for 15 min. EcTI intensified AO-stained red fluorescent-positive acidic vesicular organelles. The representative images, from three independent experiments, were observed under the same magnification, and the scale bar (25 μm) is presented in each. The impact of EcTI on SK-MEL-28 (**C**) cell morphology was analyzed through electron microscopy after 24 h of incubation; low magnification (10 µm) of control SK-MEL-28 cells ^(panel **A**)^ exhibits a typical morphology. At higher magnification (5 µm and 1 µm), the presence of healthy mitochondria ^(M)^, rough endoplasmic reticulum ^(RER)^, free poly-ribosomes ^(R)^, healthy cytoplasm ^(C)^, nucleus ^(N)^, and nuclear membrane (white arrowheads) in ^(panels **B** and **C**)^ is observed. ^(Panels **D** and **G**)^ show low magnification of EcTI-treated SK-MEL-28 cells (2 µm) presenting apoptotic bodies (long arrows) in high magnification (500 nm). ^(Panel **E**)^ and ^(panel **F**)^ show global shrinkage and disrupted cytoplasm, without affecting the nuclear membrane, which was notably intact ^(N)^. In ^(**F**)^, mitochondria in the stage of degeneration ^(M)^ are characterized by cristae disorganization (long arrows). ^(**E**, **H** and **I**)^ Autophagic vesicles (short arrows, AV), multivesicular bodies ^(MVB)^, and secretory granules ^(SG)^ were present in SK-MEL-28 EcTI-treated cells. (**D**) The impact of EcTI on the morphology of CHL-1 control cells ^(panel A)^ after 24 h of incubation, analyzed using electron microscopy at low magnification (10 µm), exhibits a typical morphology. At higher magnification (2 µm), the presence of healthy mitochondria ^(M)^, rough endoplasmic reticulum ^(RER)^, free poly-ribosomes ^(R)^, Golgi apparatus ^(GOL)^, healthy cytoplasm ^(C)^, nucleus ^(N)^, and nuclear membrane (white arrowheads) is observed in ^(panels **B** and **C**)^. In EcTI-treated CHL-1 cells, the presence of multivesicular bodies ^(MVB)^ increases in ^(panels **D** and **G**)^. In ^(panels **E** and **I**)^, apoptotic bodies are present (white long arrows) and the nuclear membrane is intact ^(N)^. The late stage of mitochondrial degeneration can be observed in ^(panel **E**, panel **G**, and panel **H**)^, where the mitochondria are mostly rounded and empty with small electron-dense masses (long arrows). EcTI-treated cells are also rich in autophagic vesicles ^(AV)^, secretory granules ^(SG)^, and multivesicular bodies ^(MVB) (panel **D**–**I**)^. Significance was considered as * *p* < 0.05, ** *p* < 0.005, and *** *p* < 0.0005.

Acidic vesicular organelles (AVOs), a typical report of autophagy, showed a significant increase at 50 and 100 µM (Figure 7A,B). Together with LC3 and BECLIN levels, Western blotting confirmed EcTI as an inducer of autophagy-associated cell death.

### 2.9. EcTI Altered Melanoma Cell Morphology

EcTI leads to morphological changes, such as membrane blebbing, mitochondrial disorganization, and disturbances of the plasma membrane, as confirmed by transmission electron microscopy of treated melanoma cells (Figure 7C—SK-MEL-28 and Figure 7D—CHL-1). This resulted in a pronounced reduction in cell quantity compared to the control. After 24 h, the remaining cells exhibited other signs of apoptosis, including structural disorganization (loss of mitochondrial cristae shape, plasma membrane disintegration, chromatin condensation, and formation of apoptotic bodies), while control cells remained unchanged. In addition, multivesicular bodies, autophagic vesicles, and secretory granules were observed.

## 3. Discussion

In general, chemotherapy is harmful to both healthy and cancerous cells. Therefore, the discovery of targeted compounds that differentiate between cancerous and non-cancerous cells is important. As previously demonstrated, EcTI did not induce significant changes in the viability of human amniotic fluid fibroblasts [10]. In this study, HUVEC and skin fibroblast viability decreased in the initial treatment but were restored by the prolonged exposure of cells to this agent. Although cell viability was partially restored after 48 h of treatment, the inhibitory effect was still valid after 72 h. The persistent detrimental effects on cell proliferation highlight their functional effectiveness.

Among the several factors that interfere with the formation of cancer, the interaction with extracellular matrix molecules is important because the expression of these molecules is increased in most tumors [20]. Thus, EcTI, which interrupts the adhesion of tumor cells to almost the entire matrix, can prevent migration and invasion, thereby preventing metastasis. Presumably, the structural features of the inhibitor provide favorable conditions for a strong interaction with adhesive proteins to interfere with the cell adhesion process. However, it should be emphasized that published data showed the effect of EcTI on gastric cancer cells without interfering with the adhesion of amniotic fluid-derived fibroblasts [9]. Similar effects were found by Bonturi and colleagues [12], where EcTI did not reduce adherence to mesenchymal stem cells derived from bone marrow tissue via collagen IV, fibronectin, and laminin, indicating that the interaction is due to the peculiar characteristics of the cell-matrix tumor.

Interference in the adhesion process of tumor cells can be reflected in proliferation, migration, invasion, and metastasis, an event in which the surrounding epithelial cells are degraded by proteolytic enzymes. This allows components of the stroma to interact with the tumor microenvironment, ensuring cancer progression and malignancy [18]. EcTI dramatically reduced the migration of both tumor cells (CHL-1, more than 80% at 5 µM; more than 70% in both cells at 100 µM). Even more interesting is its ability to intervene in the invasive process, the most effective of which was observed with CHL-1 (around 50%, 100 µM), while SK-MEL-28 was reduced by approximately 40%. These effects demonstrate the promising effects of EcTI on invasion patterns since tumor migration and invasion are essential processes in the development and metastasis of cancer. Signaling pathway proteins affected by EcTI contribute to the development of cancer and metastases, such as SRC, that promote metastasis in melanoma, FAK/SRC, whose interactions increase cell-to-cell adhesion with a consequent collective increase in cell migration [27] and PI3K protein expression. In addition, the reduction of ERK by SK-MEL-28 suggests that this pathway is responsible for the deleterious effects on the proliferation of these cells. In SK-MEL-28 cells, the reduction of ERK did not affect mTOR. On the other hand, in CHL-1 cells, EcTI decreased mTOR, suggesting that this pathway is responsible for inhibiting the proliferation, metabolism, and invasion of this cell line, indicating a difference in its mechanism of action among human malignancies.

The main difficulty of therapeutic compounds is in accessing the inside of the cancer cells [28]. This limitation does not apply to EcTI because it quickly enters the tumor cell by a mechanism that has not yet been identified. As EcTI is internalized by melanoma cells, macroscopic and microscopic cellular morphological changes provide detailed information about the mechanism by which it acts. EcTI-treated cells showed visible manifestations of cellular deadhesion, with cytoplasmic shrinkage and chromatin condensation (pyknosis), as well as nuclear fragmentation, plasma membrane blebbing, and small vesicle (apoptotic bodies) formation. The induction of cell death was also confirmed by the incorporation of propidium iodide [24], and by the decrease in its efficacy by Z-VAD and Nec-1, specific inhibitors of the apoptosis or necrosis pathway, respectively [29]. The apoptosis pathway also involved ROS induction and caspase-8 activation, which stimulated caspase-3/7, leading to cell death.

Thus, EcTI altered the metabolic state of cells by the loss of mitochondrial membrane potential [Ca^2+^], followed by the generation of ROS, which modulates cell death due to the initiation of apoptosis mediated by BAX/BCL-2 pro-apoptosis/apoptosis balancing protein. Since malignant melanoma is characterized by a poor prognosis and resistance to agents inducing apoptosis, the ability of EcTI to reduce BCL-2 and increase BAX confers on these protein-relevant characteristics to sensitize cancer cells to death.

Autophagy is another pathway that culminates in cell death and alters essential proteins of autophagosome formation. This includes the inhibition of BECLIN, the increase of ULK protein expression, and an increased LC3-II/LC3-I ratio. Upregulation of ULK may trigger increased autophagic activity by activating LC3. Confirming our previous findings, inhibition of BECLIN may result from the cleavage of caspases, such as caspase-3 and caspase-8, inducing the mitochondrial membrane permeability of cells, leading to apoptosis [25]. Interestingly, due to an increase in BECLIN, with no change in LC3-II/LC3-I expression, EcTI induces changes in the autophagic process of CHL-1 cells. Metformin, an antidiabetic drug, induces autophagy and apoptosis in melanoma cells (SK-MEL-28, G361, A375, and WM9), reduces mTOR, and increases BECLIN expression [30], a mechanism similar to our findings in the CHL-1 cells treated with EcTI.

Initially, apoptosis and necrosis were considered individual and exclusive; however, there is an interplay between apoptosis, necrosis, and autophagy as a complementary style that allows cancer cell damage [31]. The formation of AVOs is one of the morphological features of autophagy caused by EcTI, along with altered autophagic proteins (LC3 and BECLIN).

It is clear that the mechanisms involved in the harm to melanoma between the SK-MEL-28 and the CHL-1 cell lines are different, which is not surprising since they are genetically heterogeneous cells. However, in all the melanoma lines treated with EcTI, patterns of activated cell death, often by different routes, showed a non-standard effect. The cell morphology obtained by transmission electron microscopy confirmed our findings as apoptotic bodies, chromatin condensation, disorganized mitochondrial crest, active pinocytosis, formation of multivesicular bodies, and autophagic vesicles. Melanoma cells treated with EcTI showed the appearance of cell death events in different stages, with cells in the early or late apoptosis phase and autophagic vesicles, still containing sparse healthy cells resistant to the effect of the compound.

## 4. Materials and Methods

### 4.1. Plant Purification

Plant material was collected in Recife and identified by a specialist from the Instituto Agronômico de Pernambuco, where the voucher specimen (no. 61.415) was deposited. The inhibitor was purified following the procedure described by de Paula et al. [9]. The protein extracted from *E. contortisiliquum* seeds was briefly precipitated using acetone (80% (*v*/*v*), 4 °C), loaded onto a DEAE–Sepharose column in Tris/HCl (0.1 M), pH 8, and eluted with 0.15 M of NaCl added to the equilibrium buffer (Figure S1). Further purification was achieved using trypsin–Sepharose (Figure S2) and size-exclusion chromatography (Figure S3). Inhibitory activity was evaluated using a-benzoyl-D-L-arginine-r-nitroanilide (1 mM). High-performance liquid chromatography (Shimadzu, Japan, C18 column) (Figure S4) and SDS–polyacrylamide electrophoresis (Figure S5) were performed to evaluate the purity of the preparation for in vitro experiments. Total protein content was determined using the Bradford protocol [32].

### 4.2. Cell Culture

SK-MEL-28 (BRAFV600E positive) and CHL-1 human cell lines were purchased from ATCC (Alexandria, MN, USA) and cultured in high-glucose DMEM. B16F10-nex cells were generously provided by Dr. Luiz Rodolpho R. G. Travassos (Federal University of São Paulo, São Paulo, Brazil). All cells were cultured after 20–30 passages at 37 °C in a sterile humidified atmosphere with 5% CO_2_ and 95% air, supplemented with 10% fetal bovine serum (Sigma-Aldrich, Darmstadt, Germany), 100 mg/mL streptomycin, and 100 U/mL penicillin (Gibco-Thermo Fisher Scientific Inc., Waltham, MA, USA). The medium was refreshed every three days, and cells were used for experiments or cryopreservation before reaching 90% confluence. All cells previously tested negative for mycoplasma contamination (VenorTMGeM Mycoplasma Detection Kit, PCR-based, MP0025, Sigma-Aldrich) (Figure S6).

### 4.3. MTT Assay

Cell viability was evaluated by the MTT assay [33]. An aliquot of 10 mL of 3-(4,5-dimethylthiazol-2-yl)-2,5-diphenyltetrazolium bromide (5 mg/mL) was added to the cells in culture upon treatment, followed by 2 h of incubation at 37 °C. Each 96-well plate containing 100 µL/5000 SK-MEL-28, CHL-1 cells, or HUVEC and HFF-1 was preincubated for 24–72 h in the absence (control groups, 0) or presence of 5–100 µM of EcTI at 37 °C and 5% CO_2_. Then, 100 µL of DMSO was added, and the absorbance was monitored at 540 nm (Spectra Max Plus 384, Molecular Devices, San Jose, CA, USA).

To evaluate the effects of some modulators of specific types of cell death in EcTI-treated cells, Z-VAD (20 µM), necrostatin-1 (Nec-1, 5.0 µM), and necrosulfonamide (NSA, 0.2 µM) were preincubated with cells for 24 h. Then, EcTI (5–100 µM) was added and the reaction proceeded for an additional 24 h [34]. The effects of the compounds on EcTI efficacy were determined by comparison with EcTI treatment alone, which was used as a control. All experiments were performed independently in triplicate.

### 4.4. Cell Proliferation Assay

Cells (5000 cells/well) were incubated for 24 h in a 96-well plate at 37 °C and 5% CO_2_ for adherence. After initial incubation, the medium was replaced with EcTI (5–100 µM) in the experimental groups, but not in the control group. After incubation for 24 h, 10 mM BrdU was used (Cell Proliferation ELISA Chemiluminescence Kit, Thermo Fisher Scientific Inc., Waltham, MA, USA), according to the manufacturer’s instructions. A spectrophotometer (Max PLUS, Molecular Devices, San Jose, CA, USA) at 405 nm was used to measure the light emission of the samples [35].

### 4.5. Cell Adhesion

The adhesion assay was performed using collagen I (8 µg/well), collagen IV (4 µg/well), fibronectin (4 µg/well), vitronectin (0.2 µg/well), and laminin (4 µg/well) in a 96-well plate at 4 °C overnight. Approximately 5000 cells plus EcTI (0 to control, and 5–100 µM) were seeded on the attached substrates per well and incubated for 4 h at 37 °C and 5% CO_2_. Then, heat-denatured BSA (1% *w*/*v*) was added, incubated for 1 h at 37 °C, and washed with PBS buffer (137 mM NaCl, 2.7 mM KCl, 10 mM Na_2_HPO_4_, 1.8 mM KH_2_PO_4_, pH 7.4). Adherent cells were fixed using cold methanol for 40 min, washed with PBS, and stained with 1% toluidine blue (*w*/*v*) for 30 min. The cells were washed three more times with PBS and solubilized in 100 µL of 1% SDS (*w*/*v*) for 30 min at 37 °C. To measure the flattening of adherent cells, a microplate reader spectrophotometer (Spectra Max PLUS) was used to record the absorbance at 540 nm. The data are expressed as the percentage of adherent cells normalized to the percentage of the control cells [36].

### 4.6. Cell Migration

To analyze the coordinated movement of melanoma cells, 50,000 cells were seeded in a 24-well plate at 37 °C and 5% CO_2_ for adherence and scratched with a 200 µL pipette tip. Images were acquired immediately after gap creation (0 h). After the cells were washed with PBS, fresh medium containing EcTI (25, 50, and 100 µM) was added and incubated for 24 h. Images were acquired by Evosfl Inverted Digital Microscope in phase contrast, using a 4× objective lens (Sony^®^ ICX285AL CCD Camera) to assess the closure rate over time. The scratched area was analyzed using the ImageJ MRI Wound Healing Tool, and the percentages of cell migration were calculated by the wound ratio at the beginning and at the end of the wound [37]. Once the cells reached 100% confluence, the area that remained clear of cells was quantified for 24 h using the following equation:

$$\mathrm{migration}\;\left(\%\right)=\frac{\left(\mathrm{gap}\;\mathrm{closure}\;\mathrm{distance}\;\mathrm{after}\;24\;h\right)\;}{\left(\mathrm{gap}\;\mathrm{closure}\;\mathrm{distance}\;\mathrm{after}\;0\;h\right)}\times 100$$

### 4.7. Transwell Cell Invasion

Boyden chamber invasion was evaluated in 8.0 µm pore size. Melanoma cells (50,000 cells/well) were seeded onto the inserts in a 24-well plate, after polymerization with Matrigel coating (1:6 serum-free medium) for 30 min at 37 °C. Cells were pre-incubated with EcTI (5–100 µM) exclusively in the experimental groups for 15 min in a fresh, FBS-free medium. Then, 400 µL of 10% FBS medium was added to the lower chamber, followed by incubation at 37 °C and 5% CO_2_ for 24 h. The inserts were washed with PBS, and the non-invaded cells were carefully removed with a cotton swab from the upper surface of the chamber. The cells were fixed with cold methanol for 30 min and stained with 1% (*w*/*v*) toluidine blue. They were washed again in PBS, and the invaded cells were counted in at least 10 visual fields under an inverted microscope using a 10× objective lens (Leica, Camera DFC3000G and Leica Application Suite, Germany) [38]. The percentage of invaded cells was calculated as the ratio of control groups to the EcTI addition groups. The calculation of cells with invasive potential was performed as shown below:

$$\mathrm{invasion}\;\left(\%\right)=\frac{\mathrm{number}\;\mathrm{of}\;\mathrm{cells}\;\mathrm{that}\;\mathrm{invaded}\;\mathrm{after}\;\mathrm{treatment}\;\mathrm{with}\;\mathrm{EcTI}}{\left(\mathrm{number}\;\mathrm{of}\;\mathrm{cells}\;\mathrm{that}\;\mathrm{invaded}\;\mathrm{in}\;\mathrm{control}\right)}\times 100$$

### 4.8. Nuclear Morphology

Hoechst 33,342 nuclear staining (Thermo Fisher Scientific Inc., Waltham, MA, USA) was used to detect the nuclear changes. In total, 5000 cells/well were seeded in a 96-well black plate for 24 h at 37 °C and 5% CO_2_. After this period, EcTI (25, 50, and 100 µM) was added to each well and incubated for another 24 h, washed thoroughly with PBS, and incubated for 10 min with Hoechst staining solution (2 µg/mL, diluted in PBS, 100 µL/well). Nuclear staining was observed with an inverted fluorescence microscope (Leica) using a 10× objective lens and a set of blue filter cubes (excitation: 394–410 and emission: 435–485). The nuclear area (µm^2^) was measured using ImageJ software, using the Make Binary Tool and Measure. The percentages of the nuclear area were calculated using the control group compared to the EcTI treatment groups [39].

### 4.9. PI Uptake

To analyze the internalization of propidium iodide (PI), 5000 cells were seeded in a 96-well black plate for 24 h at 37 °C and 5% CO_2_. EcTI (25, 50, 100 µM) was added, and the cells were incubated for another 24 h and washed with PBS. To monitor pore formation, PI (5 µg/mL, diluted in PBS, 100 µL/well) was added. Triton X-100 (0.01%), incubated for 24 h, was used as the positive control. Images were acquired using an inverted fluorescence microscope (Leica) with a 10× objective lens and a set of red filter cubes (excitation: 542/12 nm BP and emission: 575–640 nm BP) [39].

### 4.10. Mitochondrial Membrane Potential

To quantify changes in mitochondrial membrane potential (MMP), the cell-permeant fluorescent lipophilic dye tetramethylrhodamine ethyl ester (TMRE) (Sigma-Aldrich, USA) was used in live cells [39]. In total, 5000 cells were incubated in a 96-well black plate for 24 h at 37 °C and 5% CO_2_. Then, EcTI was added (50 and 100 µM/well) and incubated for an additional 24 h. After the addition of 100 nM TMRE to the CHL-1 cells and 500 nM to the SK-MEL-28 cells, the cells were incubated for another 45 min at 37 °C and washed with PBS to replace TMRE. Images were acquired using an inverted fluorescence microscope (Leica, Germany), using a 10× objective lens and a red filter cube set (excitation: 515–560 nm and emission: 590 nm). Next, 50 µM FCCP (carbonyl cyanide 4-((trifluoromethoxy) phenylhydrazone) was incubated for 2 h at 37 °C before TMRE staining was used as a positive control. The fluorescence of the cells was calculated using ImageJ, considering CTCF = Integrated Density − (Area of selected cell × Mean fluorescence of FCCP readings), in which CTCF is corrected for the total fluorescence of the cells.

### 4.11. Ca^2+^ Measurement

The intracellular calcium level in melanoma cells was measured using the FLUO-4-AM Direct Calcium Assay Kit according to the manufacturer’s instructions (Thermo Fisher Scientific Inc., Waltham, MA, USA). In total, 5000 cells/well were incubated in a 96-well black plate for 24 h at 37 °C and 5% CO_2_. Thereafter, EcTI (25, 50, and 100 µM) was added and the mixture was incubated for another 24 h. Then, FLUO-4-AM (5 µM) was loaded and incubated for 1 h at 37 °C. Images were acquired using an inverted fluorescence microscope (Leica, Germany), using a 10× objective lens, and absorbance was read with the Spectrophotometer FlexStation Multi-Mode Microplate Reader (Molecular Devices) at 485 nm for excitation and 530 nm for emission. Data were analyzed as a relative fluorescence unit (RFU) [34,39].

### 4.12. ROS Production

CM-H_2_DCFDA, a general indicator of reactive oxygen species (ROS), was used to analyze ROS production. A total of 50,000 cells (SK-MEL-28 and CHL-1)/well were incubated in a 24-well plate for 24 h at 37 °C and 5% CO_2_. Subsequently, EcTI (50 and 100 µM/) or H_2_O_2_ (500 µM) was added to the wells for an additional 24 h incubation period. After this, cells were stained with 5 µM of the fluoroprobe CM-H_2_DCFDA for 40 min, except for the negative control (unstained cells) group. The medium was discarded. Cells were washed with PBS and collected to determine ROS production using the FL1-H channel in the BD Accuri C6 flow cytometer (BD, Los Angeles, CA, USA) and Flow Jo 10 software.

### 4.13. Caspase-3/7 Activity

For caspase-3 and -7 activity, the CellEvent^®^ Caspase-3/7 Green reagent was used according to the manufacturer’s instructions (Thermo Fisher Scientific Inc., Waltham, MA, USA). A total of 5000 cells/well were seeded into a 96-well black plate and cultivated for 24 h at 37 °C and 5% CO_2_. EcTI was added at concentrations of 25, 50, and 100 µM/for 24 h, and fresh medium was added to the control group. After this period, the fluorogenic substrate (1 µM) was loaded per well, followed by a 30 min incubation period at 37 °C. Images were acquired using an inverted fluorescence microscope (Leica, Wetzlar, Germany) using a 10× objective lens, and 494 nm for excitation and 516 nm for emission.

### 4.14. Flow Cytometry—Cell Death

To evaluate cell death, 50,000 cells were seeded in a 24-well plate for 24 h at 37 °C and 5% CO_2_ for complete adherence and treated with EcTI (5–100 µM) in the experimental groups or fresh medium in the control group. For cytometric analysis, cells were detached using Versene solution (0.2 g EDTA/liter of PBS; Thermo Fisher Scientific Inc., Waltham, MA, USA) and washed with PBS. The suspension was labeled with cell death staining solution (2.5 µg/mL annexin-FITC and 5 µg/mL PI in binding buffer (0.1 M HEPES/NaOH, pH 7.4, 1.4 M NaCl plus 25 mM CaCl_2_)) according to the manufacturer’s instructions (Annexin V/Dead Cell Apoptosis Kit, Thermo Fisher Scientific). The cells were incubated for 15 min at room temperature, protected from light, and at least 10,000 events were collected for acquisition. Dot plot analyses were performed in a BD Accuri C6 Cytometer using C6 Accuri Software (BD, Los Angeles, CA, USA). Staurosporine (1 µM) was used as the apoptosis control, and Triton X-100 (0.1%) was used as the necrosis control [40].

### 4.15. EcTI Internalization

The conjugation of EcTI to AlexaFluor488 (Thermo Fisher Scientific Inc., Waltham, MA, USA) was carried out as recommended by the manufacturer for 3 h with constant agitation. Thereafter, the solution was subjected to molecular exclusion chromatography (Superdex75 10/300GL) to remove excess unbound fluorophores. For the internalization assay, 8000 human melanoma cells were seeded into a black 96-well plate for 24 h. In sequence, cells were incubated with 12.5 µM EcTI-AlexaFluor488 for 10 and 30 min, 1, 2, and 4 h, and then washed with PHEM (2 mM HEPES, 10 mM EGTA, 2 mM MgCl_2_, 60 mM Pipes, pH 6.9). Afterwards, cells were fixed with PFA (5% (*w*/*v*) diluted in PHEM plus 5% sucrose (*w*/*v*) and 0.25% Triton X-100 (*v*/*v*)) for 15 min and washed thoroughly with PHEM containing 0.75% glycine (*w*/*v*). Cells were then stained with 5 µM Hoechst for 15 min at room temperature and washed 3 times with PHEM. Subsequently, ImageXpress Micro Confocal High-Content Screening equipment was used to analyze labeled cells with fluorescent compounds. The MetaXpress high-content image acquisition and analysis software was used to analyze EcTI internalization of at least 60 cells/field and 9 sites/well.

### 4.16. Western Blotting

SK-MEL-28 and CHL-1 (500,000 cells/well) were incubated in a 6-well plate for 24 h at 37 °C and 5% CO_2_. After the addition of EcTI (25, 50, and 100 µM), an additional 24 h incubation period occurred. Cells were then mechanically detached using RIPA buffer (50 mM Tris/HCl, pH 7.4, 150 mM NaCl, 1 mM EGTA, 1% Tween 20, 0.25% sodium deoxycholate, sodium orthovanadate, and 1 mM sodium fluoride) plus fresh phosphatase and protease inhibitors. Total lysate protein was quantified using a MicroBCA Assay Kit (Thermo Fisher Scientific Inc., Waltham, MA, USA). Next, 30 µg was loaded onto SDS-PAGE gel (10%–5%), transferred to PVDF membranes (0.2 µm) using a Trans-Blot Turbo Transfer System (Bio-Rad, Hercules, CA, USA), and blocked with BSA (2 h at room temperature) to receive primary antibodies, after washing with TBST solution (25 mM Tris/HCl, pH 8, 192 mM glycine, 0.1% Tween-20). Then, PVDF membranes were incubated overnight, at 4 ºC, with primary antibodies, diluted 1:1000 (purchased from Cell Signaling, except phospho-PI3K from BD): anti-FAK (#3285), anti-phospho FAK (#8556, Y397), anti-SRC (#2109), anti-phospho SRC (#2101, Y416), anti-PI3K (#BD 610045), anti-phospho PI3K (#4228 Y458/Y199), anti-MTOR (#2983), anti-phospho MTOR (#2971 S2448), anti-ERK (#4695), anti-phospho ERK (#4370 T202/Y204), anti-BCL-2 (#2876), anti-BAX (#2772), anti-BECLIN (#3495), anti-ULK (#8054), anti-AMBRA1 (#24907), anti-LC3A/B (#12741), and anti-GAPDH (#5174). Thereafter, membranes were incubated for 1 h at room temperature with secondary antibodies, anti-rabbit (#7074) or anti-mouse (#7076), diluted 1:1000, detected by chemiluminescence (West Dura Extended, Thermo Fisher Scientific Inc., Waltham, MA, USA) using the Gel System Uvitec—Alliance 4.7. Densitometric analysis was performed using ImageG software, with GAPDH as a control for each sample, and the phosphorylated protein to total protein ratio was calculated after GAPDH normalization.

### 4.17. Detection of Acidic Vesicular Organelles

To further study the autophagy process, we evaluated the formation of acidic vesicular organelles, a morphological feature of autophagy [41]. An acidic cell compartment was detected by the metachromatic dye acridine orange (AO). SK-MEL-28 and CHL-1 cells (50,000 cells/well) were seeded in a 24-well plate before treatment with EcTI (50 µM and 100 µM for 24 h) or EBSS medium (for 3 h, positive control). The cells were then stained with AO (0.5 μg/mL) for 15 min. The samples were observed using a fluorescence microscope with excitation at 488 nm. Green (emission: 530–550 nm) and red (emission: 650 nm) fluorescence were examined using a 40× objective lens (Leica, Wetzlar, Germany).

### 4.18. Transmission Electron Microscopy

Cells (50,000 cells/well) were seeded in a 24-well plate before treatment with EcTI (100 µM) for 24 h, washed with PBS, and incubated with Versene solution for 5–7 min. Cells were fixed with 2% (*v*/*v*) formaldehyde and 2.5% (*v*/*v*) glutaraldehyde for 30 min at room temperature and diluted in the buffer cacodylate (0.1 M, pH 7.2). Cells were then embedded in 4% (*v*/*v*) agar, and fixed with 2% osmium tetroxide in sodium cacodylate (0.1 M, pH 7.2) for 2 h. Then, the cells were dehydrated in increasing ethanol concentrations (70%, 90% (*v*/*v*), and absolute). A mixture of propylene oxide and Epon 612 resin (1:1) was added to the samples overnight. Then, pure Epon was added to the mixture for an additional 2 h under vacuum and polymerized in an oven at 60 °C for 48 h. In the fenestrated copper metallic grid, ultra-thin cuts (70 nm) were placed, and uranyl acetate/plumb citrate was used as a contrast. The images were examined using transmission electron microscopy (TEM, JEOL JEM 1200 EX II, JEOL, Peabody, MA, USA) operated at 80 kV. Micrographs were acquired with a GATAN 791 camera (USA), while brightness, contrast levels, and image resolution were adjusted using ADOBE Photoshop 7.

### 4.19. Statistical Analysis

All experiments were performed in triplicate and independently repeated at least thrice. Data are expressed as the mean ± standard deviation (SD), and statistical analyses were performed using GraphPad Prism version 8 (GraphPad Software, San Diego, CA, USA). Comparisons between experimental groups were conducted using one-way ANOVA followed by Tukey’s test. Statistical significance is indicated by * *p* < 0.05, ** *p* < 0.005, and *** *p* < 0.0005.

## 5. Conclusions

**Figure 8:**
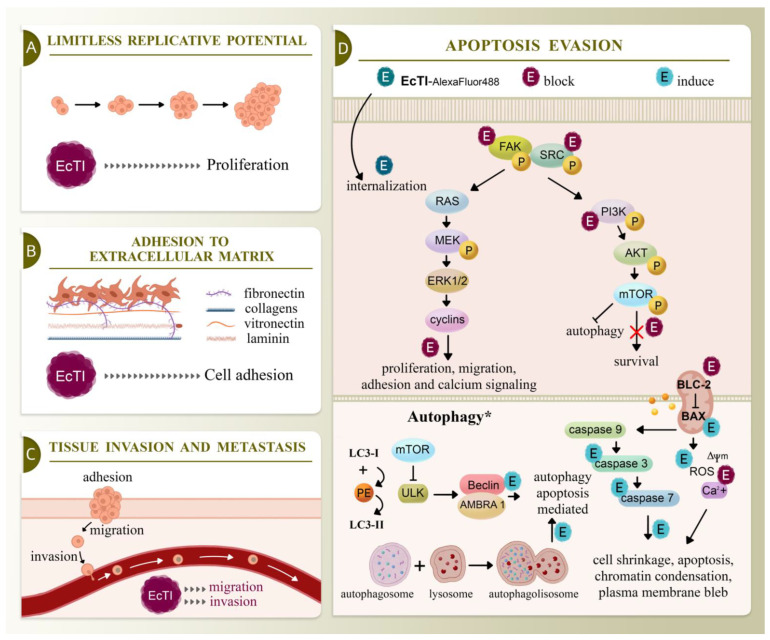
Signaling pathways of cancers include limitless replicative potential, adhesion to the extracellular matrix, tissue invasion and metastasis, and apoptosis evasion. (**A**) EcTI inhibits the replicative potential of melanoma cells, interfering with components of the extracellular matrix and reducing melanoma cell adhesion (**B**), which blocks melanoma migration, invasion, and metastasis (**C**). These events are mediated by the impairment in apoptosis evasion, interfering with proteins associated with proliferation, migration, adhesion, and calcium (Ca^2+^) signaling and those associated with the autophagy/apoptosis pathways, leading to cell shrinkage, chromatin condensation, plasma membrane blebbing, reactive oxygen species production (ROS), mitochondrial membrane potential disruption (Δ**ψ**_m_), and apoptosis of melanoma cells (**D**). These findings together indicate the deleterious effect of EcTI on the SK-MEL-28 and CHL-1 cell lines by distinct pathways.

With EcTI treatment, a sustained response against human melanoma cells has been observed by inhibiting FAK, SRC, and PI3K proteins and reducing functions essential for tumor regulation, such as cell proliferation, migration, invasion, and cell adhesion to multiple matrix molecules commonly upregulated in melanoma. EcTI still modified cell morphology, as observed by pyknosis and nuclear condensation, in addition to enhancing PI uptake, suggesting a pattern of cell death. In addition, EcTI increased caspase-3/7 activity, which altered the mitochondrial transmembrane potential and intracellular calcium concentration, stimulated cell death, which was also observed by marked BAX/BCL-2 expression, and generated ROS. This apoptotic/autophagic evidence was confirmed by electron microscopy encompassing non-canonical signaling pathways involving autophagic proteins such as ULK and LC3-II/LC3-I with amplified expression in SK-MEL-28 cells. Conversely, in CHL-1, EcTI stimulated BECLIN expression, pointing to the differences in the mechanism of action between these human cell lineages (Figure 8).

Taken together, our studies have shown that in addition to the inhibition of proteolytic enzymes, plant inhibitors can be used to block cancer-related events, suggesting the potential of this protein in the investigation of melanoma skin cancer. Even with some questions posed in carrying out this study, and with the awareness that there is much to be investigated further, this work aims to contribute to the understanding of EcTI activity, evaluate the relationship of its functional properties and mechanisms of action, and encourage further studies on the development of promising compounds for melanoma.

## Data Availability

Not applicable.

## Supplementary Materials

The following supporting information can be downloaded at: https://www.mdpi.com/article/10.3390/molecules27092956/s1, Figure S1: Ion-exchange chromatography on DEAE–Sepharose of EcTI; Figure S2: Trypsin–Sepharose chromatography of EcTI; Figure S3: Molecular exclusion chromatography of EcTI; Figure S4: High-performance liquid chromatography of EcTI; Figure S5: Electrophoresis gel (representative fractions of the EcTI isolation steps); Figure S6: Analysis of mycoplasma in melanoma cells; Figure S7: Cell viability by Presto Blue; Figure S8: Melanoma cell morphology; Figure S9: ROS production; Figure S10: Apoptosis of human melanoma cells treated with EcTI for 24 h.

## Sample Availability

Samples of the compounds are not available from the authors.

## References

[1] Desai A.G., Qazi G.N., Ganju R.K., El-Tamer M., Singh J., Saxena A.K., Bedi Y.S., Taneja S.C., Bhat H.K. (2008). Medicinal Plants and Cancer Chemoprevention. Curr. Drug Metab..

[2] Greenwell M., Rahman P. (2015). Medicinal Plants: Their Use in Anticancer Treatment. Int. J. Pharm. Sci. Res..

[3] Zanrè V., Campagnari R., Cerulli A., Masullo M., Cardile A., Piacente S., Menegazzi M. (2022). Salviolone from *Salvia miltiorrhiza* Roots Impairs Cell Cycle Progression, Colony Formation, and Metalloproteinase-2 Activity in A375 Melanoma Cells: Involvement of P21(Cip1/Waf1) Expression and STAT3 Phosphorylation. Int. J. Mol. Sci..

[4] Ruzzolini J., Peppicelli S., Andreucci E., Bianchini F., Scardigli A., Romani A., la Marca G., Nediani C., Calorini L. (2018). Oleuropein, the Main Polyphenol of Olea europaea Leaf Extract, Has an Anti-Cancer Effect on Human BRAF Melanoma Cells and Potentiates the Cytotoxicity of Current Chemotherapies. Nutrients.

[5] He Y., Li W., Hu G., Sun H., Kong Q. (2018). Bioactivities of EF24, a Novel Curcumin Analog: A Review. Front. Oncol..

[6] Kobayashi H., Suzuki M., Kanayama N., Terao T. (2004). A soybean Kunitz trypsin inhibitor suppresses ovarian cancer cell invasion by blocking urokinase upregulation. Clin. Exp. Metastasis.

[7] Roversi F.M., Saad S.T.O., Machado-Neto J.A. (2018). Serine peptidase inhibitor Kunitz type 2 (SPINT2) in cancer development and progression. Biomed. Pharmacother..

[8] Ranasinghe S.L., Rivera V., Boyle G., McManus D. (2019). Kunitz type protease inhibitor from the canine tapeworm as a potential therapeutic for melanoma. Sci. Rep..

[9] Zhou D., Lobo Y.A., Batista I.F.C., Marques-Porto R., Gustchina A., Oliva M.L.V., Wlodawer A. (2013). Crystal Structures of a Plant Trypsin Inhibitor from *Enterolobium contortisiliquum* (EcTI) and of Its Complex with Bovine Trypsin. PLoS ONE.

[10] de Paula C.A.A., Coulson-Thomas V.J., Ferreira J.G., Maza P.K., Suzuki E., Nakahata A.M., Nader H.B., Sampaio M.U., Oliva M.L.V. (2012). *Enterolobium contortisiliquum* Trypsin Inhibitor (EcTI), a Plant Proteinase Inhibitor, Decreases in Vitro Cell Adhesion and Invasion by Inhibition of Src Protein-Focal Adhesion Kinase (FAK) Signaling Pathways. J. Biol. Chem..

[11] Nakahata A.M., Mayer B., Ries C., de Paula C.A.A., Karow M., Neth P., Sampaio M.U., Jochum M., Oliva M.L.V. (2011). The effects of a plant proteinase inhibitor from Enterolobium contortisiliquum on human tumor cell lines. Biol. Chem..

[12] Bonturi C.R., Motaln H., Silva M.C.C., Salu B.R., De Brito M.V., Costa L.D.A.L., Torquato H.F.V., Nunes N.N.D.S., Paredes-Gamero E.J., Turnšek T.L. (2018). Could a plant derived protein potentiate the anticancer effects of a stem cell in brain cancer?. Oncotarget.

[13] Lobo Y.A., Bonazza C., Batista F.P., Castro R.A., Bonturi C.R., Salu B.R., Sinigaglia R.D.C., Toma L., Vicente C.M., Pidde G. (2020). EcTI impairs survival and proliferation pathways in triple-negative breast cancer by modulating cell-glycosaminoglycans and inflammatory cytokines. Cancer Lett..

[14] Cummings B.S., Schnellmann R.G. (2021). Measurement of Cell Death in Mammalian Cells. Curr. Protoc..

[15] Dunai Z.A., Imre G., Barna G., Korcsmáros T., Petak I., Bauer P.I., Mihalik R. (2012). Staurosporine Induces Necroptotic Cell Death under Caspase-Compromised Conditions in U937 Cells. PLoS ONE.

[16] Zhao X., Guan J.-L. (2011). Focal adhesion kinase and its signaling pathways in cell migration and angiogenesis. Adv. Drug Deliv. Rev..

[17] Gkretsi V., Stylianopoulos T. (2018). Cell Adhesion and Matrix Stiffness: Coordinating Cancer Cell Invasion and Metastasis. Front. Oncol..

[18] Miyazaki K., Oyanagi J., Hoshino D., Togo S., Kumagai H., Miyagi Y. (2019). Cancer cell migration on elongate protrusions of fibroblasts in collagen matrix. Sci. Rep..

[19] Galluzzi L., Vitale I., Aaronson S.A., Abrams J.M., Adam D., Agostinis P., Alnemri E.S., Altucci L., Amelio I., Andrews D.W. (2018). Molecular mechanisms of cell death: Recommendations of the Nomenclature Committee on Cell Death 2018. Cell Death Differ..

[20] Hanahan D., Weinberg R.A. (2011). Hallmarks of cancer: The next generation. Cell.

[21] Orrenius S., Gogvadze V., Zhivotovsky B. (2015). Calcium and mitochondria in the regulation of cell death. Biochem. Biophys. Res. Commun..

[22] Briston T., Roberts M., Lewis S., Powney B., Staddon J.M., Szabadkai G., Duchen M.R. (2017). Mitochondrial permeability transition pore: Sensitivity to opening and mechanistic dependence on substrate availability. Sci. Rep..

[23] Suski J., Lebiedzinska M., Bonora M., Pinton P., Duszynski J., Wieckowski M.R. (2018). Relation Between Mitochondrial Membrane Potential and ROS Formation. Methods Mol. Biol..

[24] Vermes I., Haanen C., Reutelingsperger C. (2000). Flow cytometry of apoptotic cell death. J. Immunol. Methods.

[25] Kale J., Osterlund E.J., Andrews D.W. (2018). BCL-2 family proteins: Changing partners in the dance towards death. Cell Death Differ..

[26] Chen Q., Kang J., Fu C. (2018). The independence of and associations among apoptosis, autophagy, and necrosis. Signal Transduct. Target. Ther..

[27] Lee J.J., van de Ven R.A.H., Zaganjor E., Ng M.R., Barakat A., Demmers J.J.P.G., Finley L.W.S., Herrera K.N.G., Hung Y.P., Harris I.S. (2018). Inhibition of epithelial cell migration and Src/FAK signaling by SIRT3. Proc. Natl. Acad. Sci. USA.

[28] Zhang R., Qin X., Kong F., Chen P., Pan G. (2019). Improving cellular uptake of therapeutic entities through interaction with components of cell membrane. Drug Deliv..

[29] Pereira F., Arruda D., Figueiredo C., Massaoka M., Matsuo A., Bueno V., Rodrigues E. (2013). FTY720 induces apoptosis in B16F10-NEX2 murine melanoma cells, limits metastatic development in vivo, and modulates the immune system. Clinics.

[30] Tomic T., Botton T., Cerezo M., Robert G., Luciano F., Puissant A., Gounon P., Allegra M., Bertolotto C., Bereder J.-M. (2011). Metformin inhibits melanoma development through autophagy and apoptosis mechanisms. Cell Death Dis..

[31] Nikoletopoulou V., Markaki M., Palikaras K., Tavernarakis N. (2013). Crosstalk between apoptosis, necrosis and autophagy. Biochim. Biophys. Acta.

[32] Bradford M.M. (1976). A rapid and sensitive method for the quantitation of microgram quantities of protein utilizing the principle of protein-dye binding. Anal. Biochem..

[33] Mosmann T. (1983). Rapid colorimetric assay for cellular growth and survival: Application to proliferation and cytotoxicity assays. J. Immunol. Methods.

[34] Paredes-Gamero E.J., Martins M.N., Cappabianco F.A., Ide J., Miranda A. (2012). Characterization of dual effects induced by antimicrobial peptides: Regulated cell death or membrane disruption. Biochim. Biophys. Acta..

[35] Padet L., St-Amour I., Aubin E., Proulx D.P., Bazin R., Lemieux R. (2009). Dose-Dependent Inhibition of BrdU Detection in the Cell Proliferation ELISA by Culture Medium Proteins. J. Immunoass. Immunochem..

[36] Humphries M.J. (2009). Cell Adhesion Assays. Methods Pharmacol. Toxicol..

[37] Justus C.R., Leffler N., Ruiz-Echevarria M., Yang L.V. (2014). In vitro Cell Migration and Invasion Assays. J. Vis. Exp..

[38] Jonkman J., Cathcart J.A., Xu F., Bartolini M.E., Amon J.E., Stevens K.M., Colarusso P. (2014). An introduction to the wound healing assay using live-cell microscopy. Cell Adhes. Migr..

[39] Da Silva A.M.B., Silva-Gonçalves L.C., Oliveira F.A., Arcisio-Miranda M. (2018). Pro-necrotic Activity of Cationic Mastoparan Peptides in Human Glioblastoma Multiforme Cells Via Membranolytic Action. Mol. Neurobiol..

[40] Rieger A.M., Nelson K.L., Konowalchuk J.D., Barreda D.R. (2011). Modified Annexin V/Propidium Iodide Apoptosis Assay for Accurate Assessment of Cell Death. J. Vis. Exp..

[41] Shin S.W., Kim S.Y., Park J.-W. (2012). Autophagy inhibition enhances ursolic acid-induced apoptosis in PC3 cells. Biochim. Biophys. Acta.

