# Supplementary material for: Proliferation and Invasion of Melanoma Are Suppressed by a Plant Protease Inhibitor, Leading to Downregulation of Survival/Death-Related Proteins
Source: Molecules. 2022 May 5;27(9):2956. doi: 10.3390/molecules27092956 (PMC9104945; doi:10.3390/molecules27092956)
Supplement: Supplementary file 1 [file molecules-27-02956-s001.zip › molecules-1668958-supplementary.pdf]

Supplementary Materials

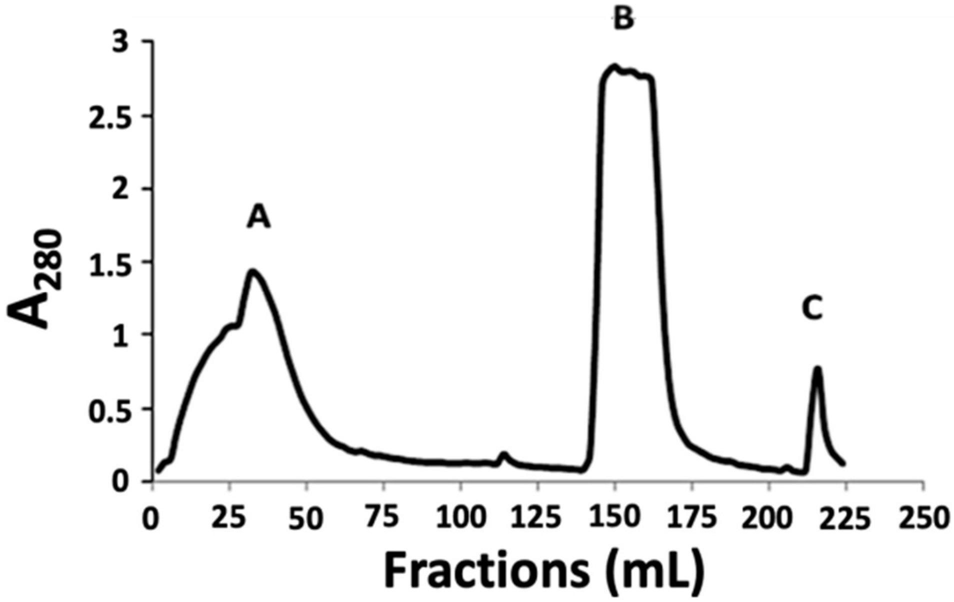

Figure S1. Ion-exchange chromatography on DEAE-Sepharose of EcTI. The resin (5 mL) was equilibrated with 0.1 M Tris/HCl buffer, pH 8.0. Sample: 150 mg of protein (A280) from acetone fractionation (80%, v/v) solubilized with 0.1 M Tris/HCl buffer, pH 8.0. (**A**) non-bounded fraction eluted with 0.1 M Tris/HCl, pH 8, (**B**) EcTI fraction eluted in 0.1 M Tris/HCl, pH 8, containing 0.15 M NaCl (**C**) unspecific proteins eluted with 0.1 M Tris/HCl buffer, pH 8, containing 0.3 M NaCl. Fractions of 1 mL were collected under a constant of 1 mL/min flow. The solid black line is the 280 nm reading. The inhibitory activity on trypsin was detected in fraction (**B**).

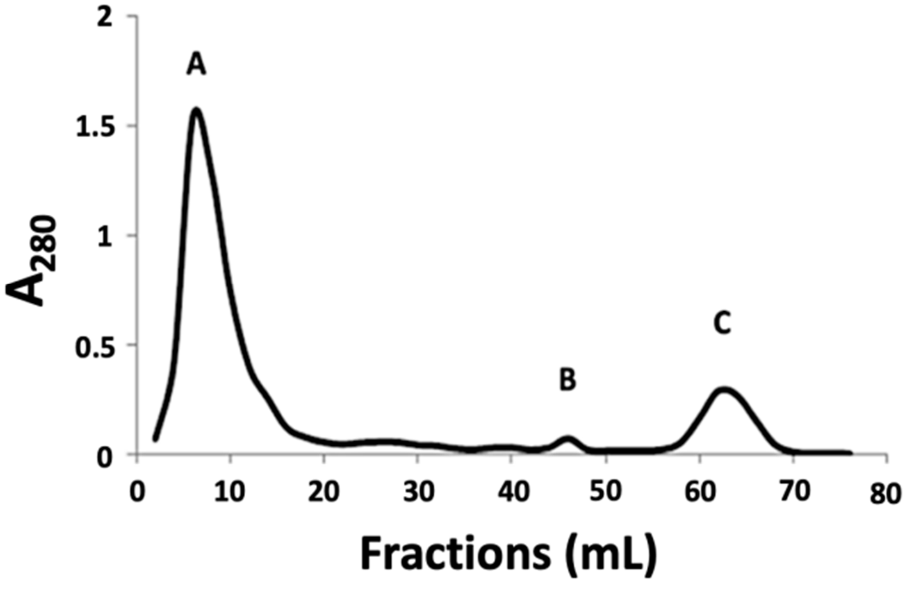

Figure S2. Trypsin-Sepharose Chromatography of EcTI. Sample: EcTI from DEAE-Sepharose eluted with Tris/HCl 0.1 M, pH 8, containing 0.15 M NaCl was applied into Trypsin-Sepharose. Equilibrium buffer: (**A**) Tris/HCl 0.1 M, pH 8, containing 0.15 M NaCl and Elution buffer: (**B**) Tris/HCl 0.1 M, pH 8, containing 0.3 M NaCl (unspecific proteins) and (**C**) KCl/HCl 0.5 M, pH 2.0 with subsequent neutralization with 50 μL of 1 M Tris/HCl buffer, pH 9.0 – EcTI fraction. The solid black line is the 280 nm reading. Fractions of 1 mL.

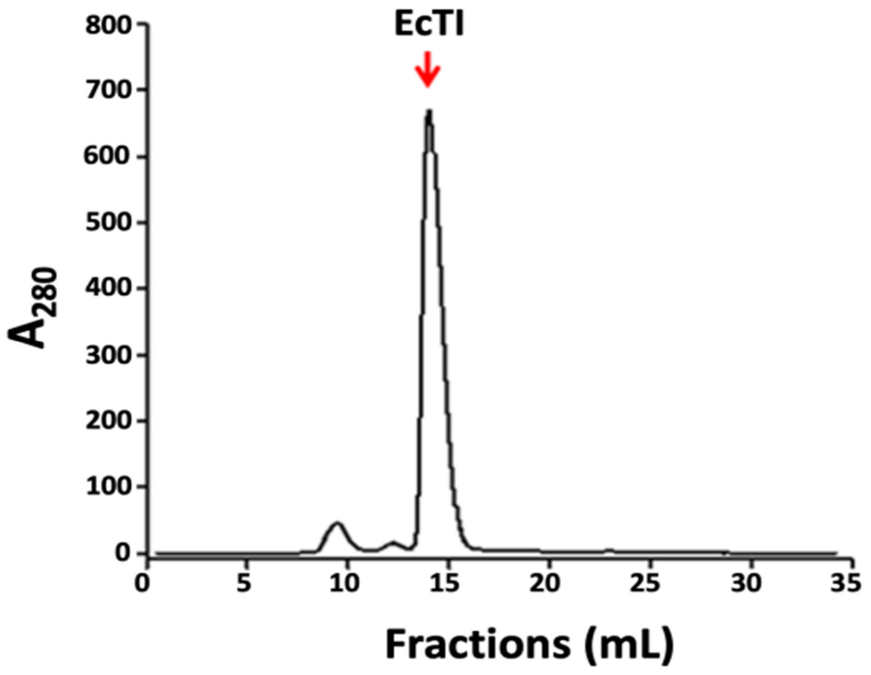

Figure S3. Molecular Exclusion Chromatography of EcTI. Sample: EcTI from Trypsin-Sepharose eluted KCl/HCl 0.5 M, pH 2.0, with subsequent neutralization with 50 µL of 1 M Tris/HCl buffer, was applied into Superdex-75 10/300 GL. The column was equilibrated with Tris/HCl 0.05 M buffer, pH 8, plus NaCl 0.15 M coupled to an ÄKTA Avant 25 (GE Healthcare). The EcTI fraction was pooled, dialyzed, lyophilized, and applied on a High-Performance Liquid Chromatography into a C18 column. The solid black line is the 280 nm reading. Fractions of 1 mL were collected under a constant flow rate of 0.5 mL/min and 1.8 MPa maximum column pressure.

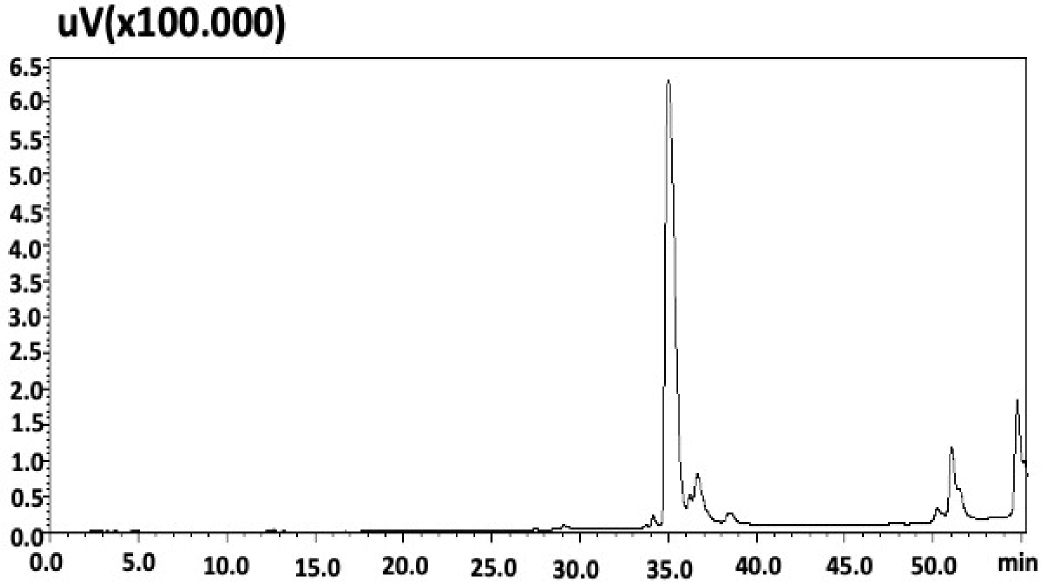

Figure S4. High-Performance Liquid Chromatography of EcTI. The fraction obtained by molecular exclusion chromatography was submitted to reverse phase chromatography on a C18 HPLC column (15 cm × 4.6 mm; Vydac). The protein fraction was eluted with a linear gradient (5–100%) of 90% (v/v) acetonitrile in 0.1% (v/v) TFA in milli-Q water at the flow rate of 0.7 mL/min (t=0.1 min, 5% B; t=5 min, 5% B; t=30 min, 40% B; t=50 min, 50% B; t=60 min, 100% B; t=65–68 min, 0% B).

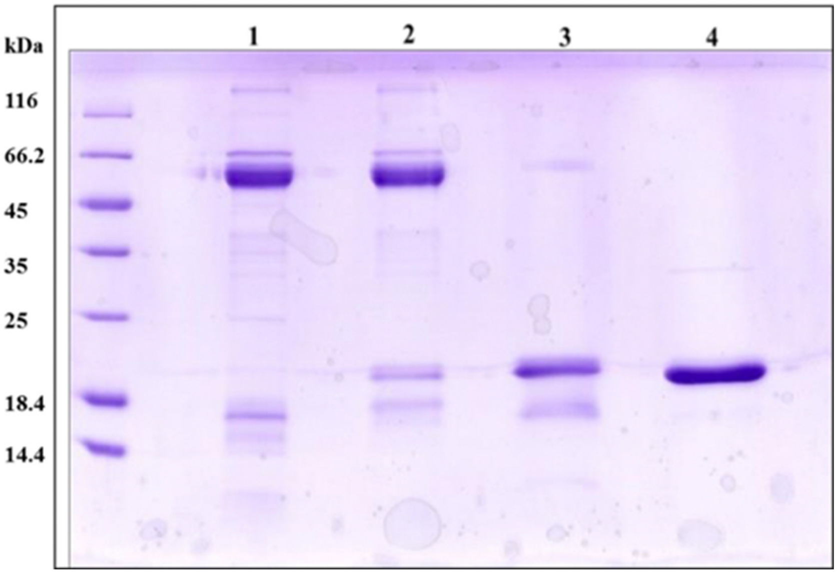

Figure S5. Electrophoresis gel (representative fractions of the EcTI isolation steps) (5% concentration gel and 12% separation gel), with (1) acetone precipitation 80% (v/v); (2) fraction obtained with Tris/HCl 0.1 M, pH 8, plus 0.15 M NaCl in DEAE-Sepharose; (3) fraction obtained with 0.5 M KCl/HCl, pH 2.0, Trypsin-Sepharose; (4) fraction obtained with Tris/HCl 0.05 M, pH 8, plus 0.15 M NaCl in Superdex-75 10/300 GL gel filtration column. All fractions have the same amount of protein (30 μg).

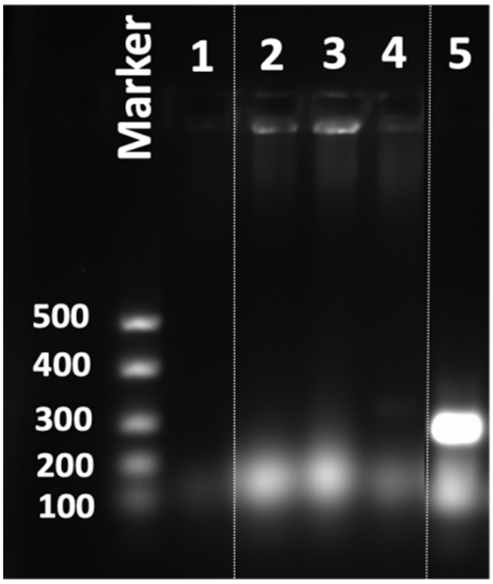

Figure S6. Analysis of mycoplasma in melanoma cells. Marker: 100 bp ladder, (**1**) negative control: deionized, DNA-free water, (**2**) SK-MEL-28 supernatant, (**3**) CHL-1 supernatant, (**4**) B16F10-nex2 supernatant, and (**5**) positive control (~270 bp bands). PCR analysis and 1.5 % standard agarose gel run were followed as specifications recommended by the manufacturer (VenorTMGeM Mycoplasma Detection Kit, PCR-based, MP0025, Sigma-Aldrich). The dashes white lines represent a crop in the original image to center an area of interest containing only melanoma cells since PCR analysis was tested with other supernatant lineages.

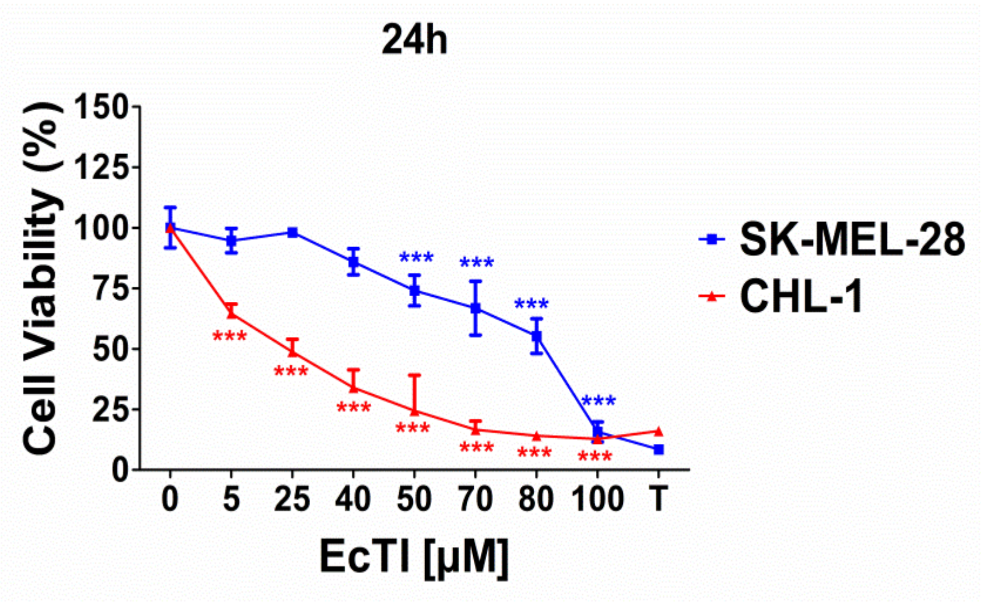

Figure S7. Cell Viability by Presto Blue. Effects of EcTI on the cell viability of melanoma cells – SK-MEL-28 and CHL-1 as measured with increased concentrations of EcTI (5, 25, 40, 50, 70, 80, and 100 μM) after 24 h. After 10 min of incubation at 37 °C, Presto Blue assay was measured using excitation/emission:535-560⁄590-615 filter in a fluorescence reader (Microplate Reader, Molecular Devices). The fluorescence values were normalized to the control in each experiment. T: Triton X-100 0.1% (v/v). The error bars represent SDs and significance reflected: * $p < 0.05$, ** $p < 0.005$ and *** $p < 0.0005$.

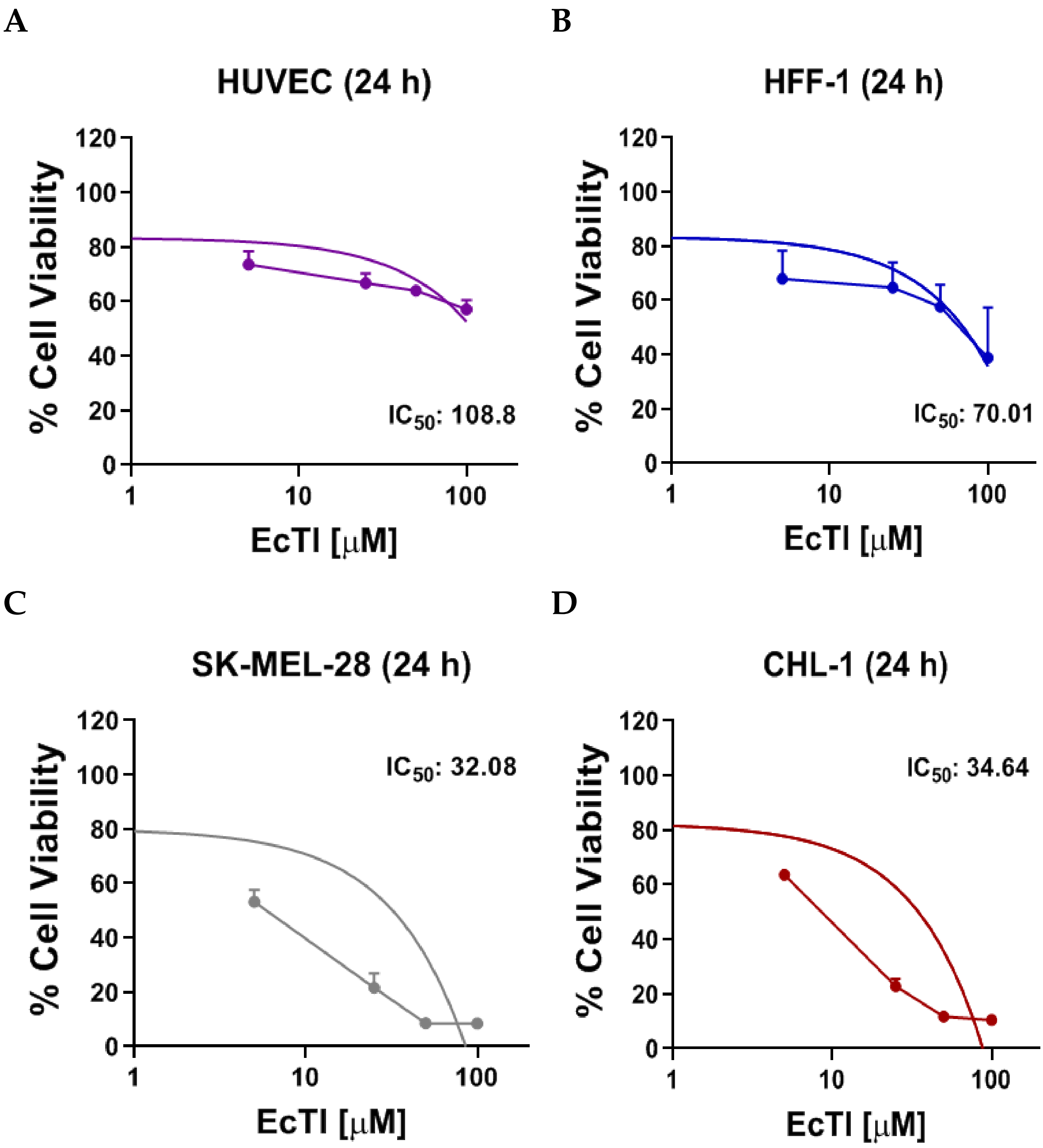

E

		SI	
**Cell Line**	**IC_{50} (μM)**	**SK-MEL-28**	**CHL-1**
**HUVEC**	108.8	3.39	3.14
**HFF-1**	70.01	2.18	2.02
**SK-MEL-28**	32.08		
**CHL-1**	34.64		

Figure S8. The concentration of a drug that is required for 50% inhibition *in vitro* (IC_{50}) of non-tumorigenic HUVEC (**A**), HFF-1 cells (**B**), and SK-MEL-28 (**C**), CHL-1 (**D**) melanoma cells after 24 h of EcTI treatment. In (**E**) selectivity index (SI) considering the ratio of non-tumorigenic (HUVEC or HFF-1) / / tumorigenic cells (SK-MEL-28 or CHL-1).

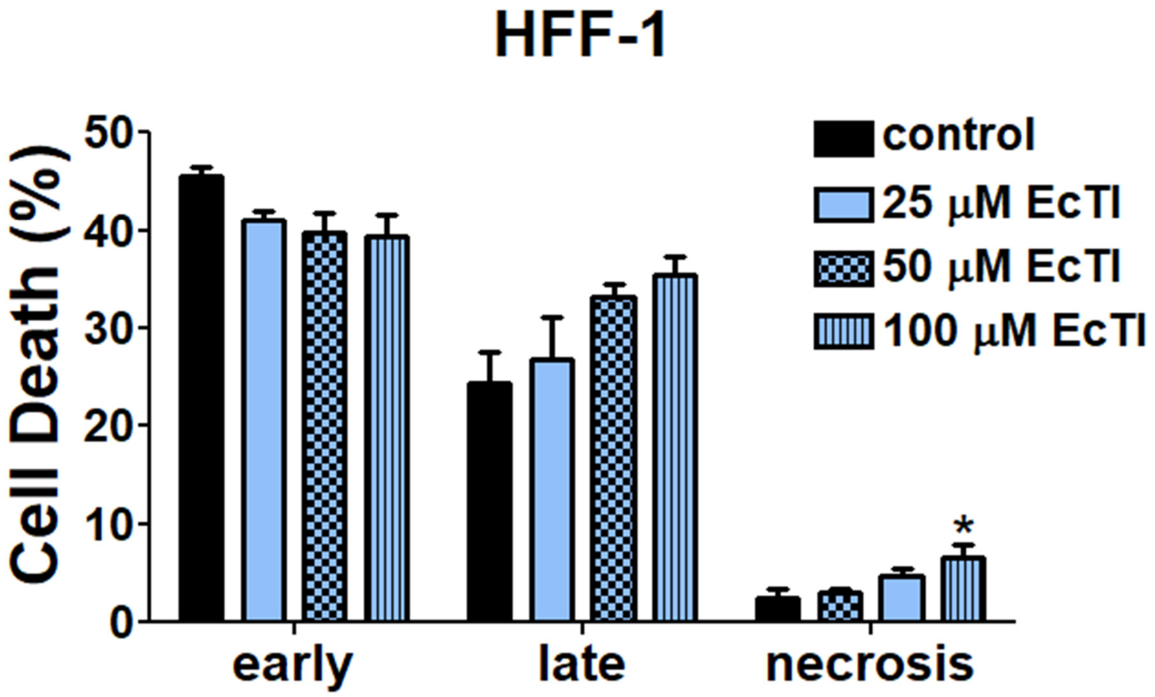

Figure S9. Cell death assay in HFF-1 (non-tumorigenic cell) after EcTI treatment for 24 h, investigated by flow cytometry, using fluorophores annexin-FITC and propidium iodide. Early and late represent stages of apoptosis. The bars represent means and SDs. Significance was considered as $^*p < 0.05$ and 10,000 events were collected for acquisition in a BD Accuri C6 Cytometer using C6 Accuri Software (BD, California, USA).

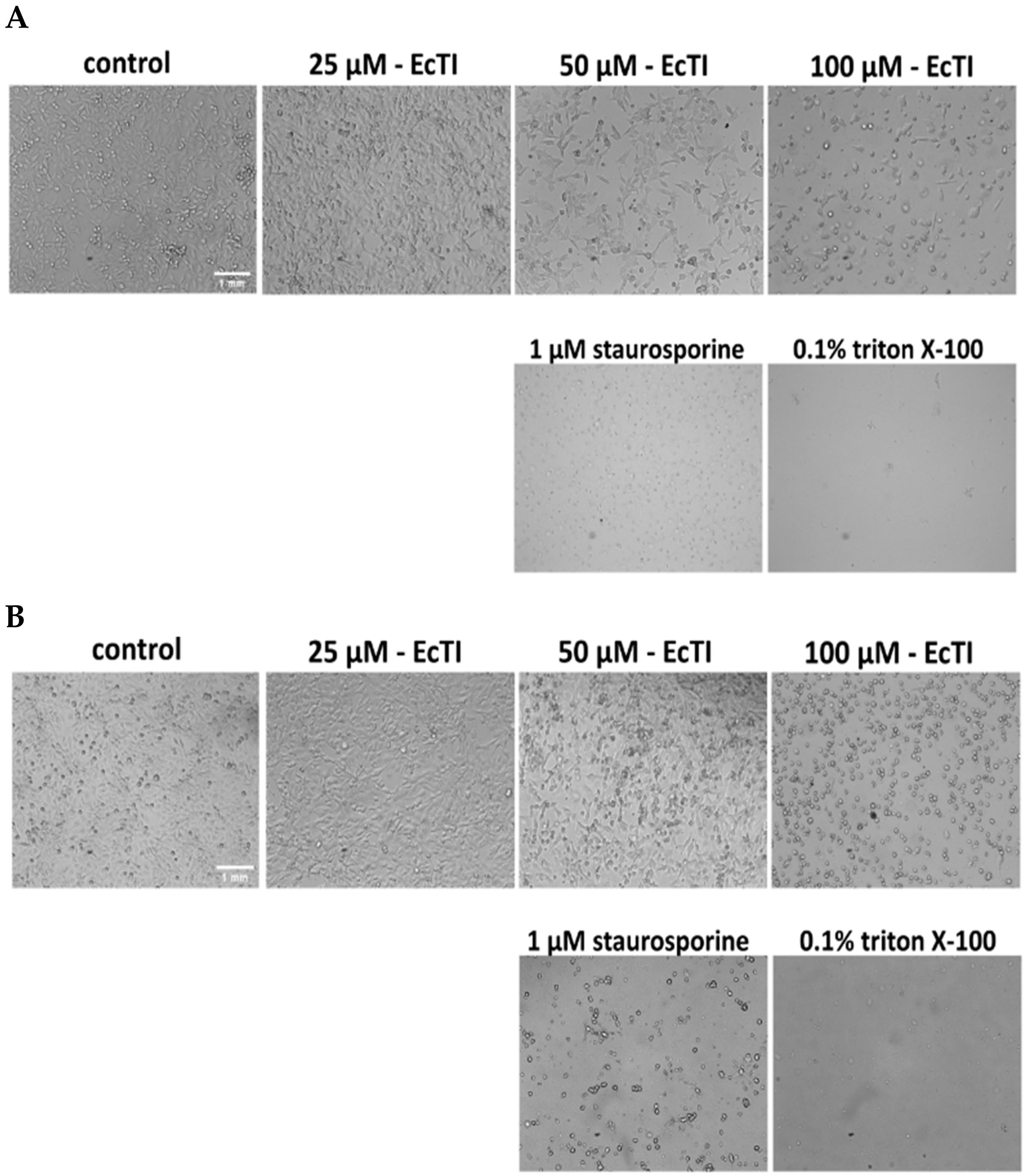

Figure S10. Melanoma Cell Morphology. EcTI induced cell detachment in 100 µM after 24 h in both human melanoma cells: (**A**) SK-MEL-28 (**B**) CHL-1. Scale bar: 1 mm. Staurosporine and triton X-100 0,1% (v/v) were used as positive controls.

A

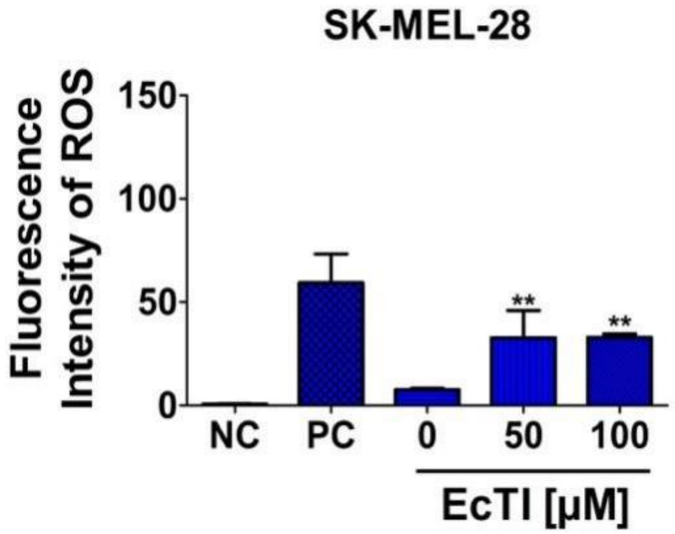

B

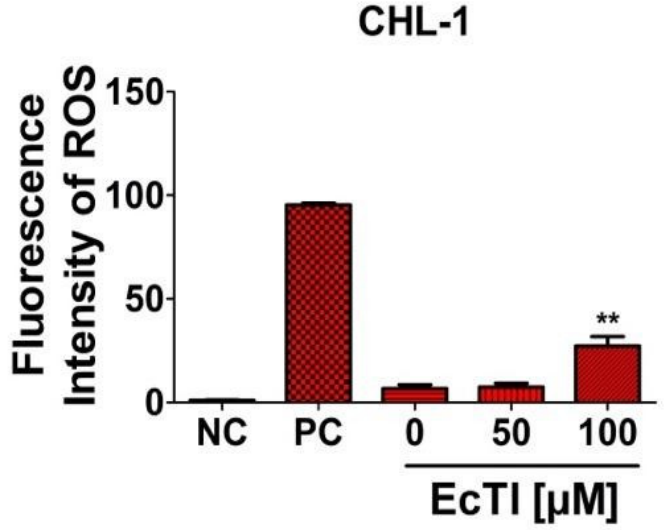

Figure S11. ROS Production. Intracellular reactive oxygen species (ROS) production was measured by 2,7-dichlorodihydrofluorescein diacetate (H2-DCFDA). EcTI increased ROS production in both, SK-MEL-28 cells (**A**) and CHL-1 cells (**B**). The fluorescence intensity of ROS was quantified by the histograms in flow cytometry (FL1-H positive) and compared to control ("0"). NC: negative control (unstained cells) and PC: positive control (500 µM of H_2O_2). The bars represent means and SDs. Significance was considered as * $p < 0.05$, ** $p < 0.005$ and *** $p < 0.0005$.

A

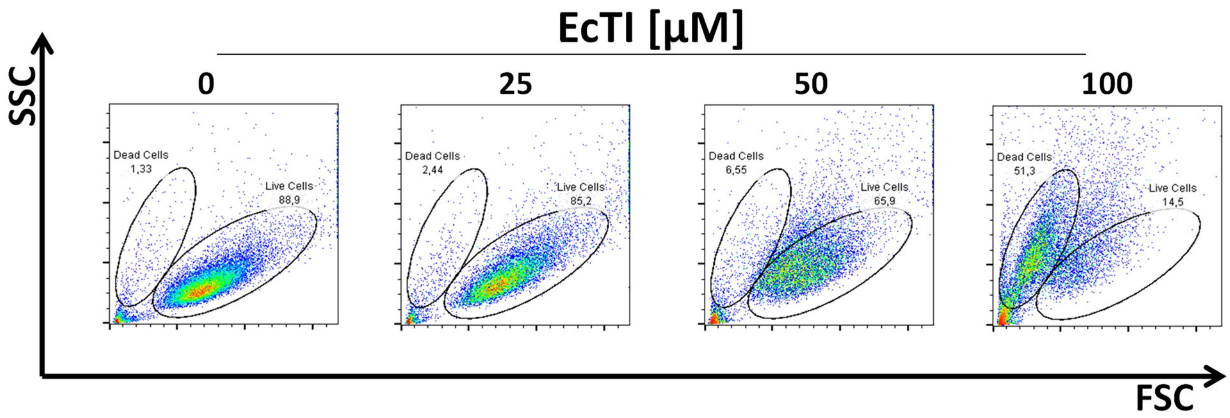

B

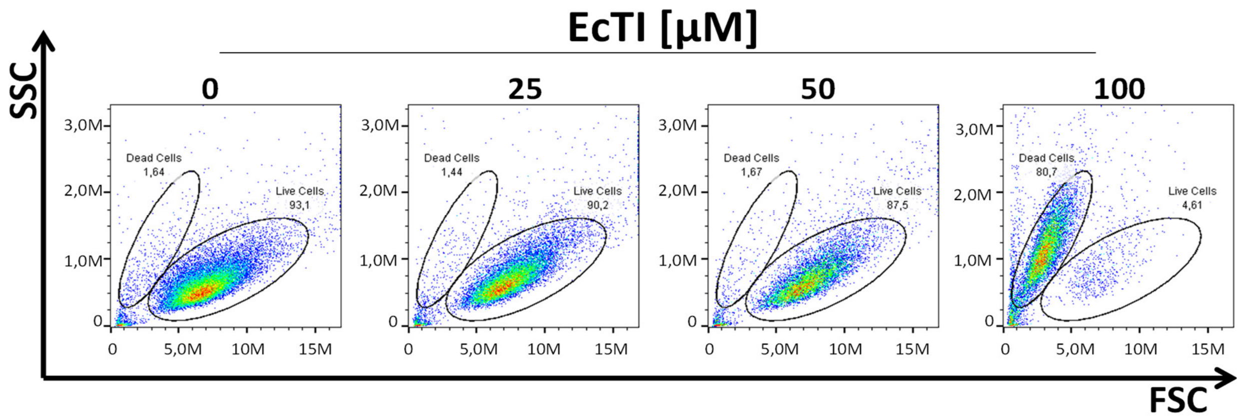

Figure S12. Apoptosis of human melanoma cells treated with EcTI for 24 h. Harvested melanoma cells (**A** – SK-MEL-28, **B** – CHL-1) were measured by side scattering (SSC, granularity) and forward scattering (FSC, size) by flow cytometry. Compared to control, representative images exemplify live/viable cells and dead cells after 25, 50, and 100 µM of EcTI (“0”).
